# ﻿Two centuries of *Pachyrhynchus* (Curculionidae, Entiminae, Pachyrhynchini) research: a comprehensive annotated checklist of taxonomy, species groups, distribution, and ecological insights in Southeast Asia

**DOI:** 10.3897/zookeys.1243.143198

**Published:** 2025-06-30

**Authors:** Yun Ho, Shan-Min Chen, Ace Kevin S. Amarga, Jing-Fu Tsai, Bin-Hong Ho, Ming-Luen Jeng, Hui-Yun Tseng

**Affiliations:** 1 Department of Entomology, National Taiwan University, No. 1, Sec. 4, Roosevelt Rd., Da’an Dist., Taipei City 106, Taiwan National Taiwan University Taipei Taiwan; 2 Biodiversity Program, Taiwan International Graduate Program, Biodiversity Research Center, Academia Sinica, Nangang District, Taipei 11529, Taiwan Biodiversity Research Center, Academia Sinica Taipei Taiwan; 3 School of Life Science, National Taiwan Normal University, Gongguan Campus, Wenshan District, Taipei 11677, Taiwan National Taiwan Normal University Taipei Taiwan; 4 Department of Biology, National Museum of Natural Science, No.1, Guanqian Rd., North Dist., Taichung City 404023, Taiwan National Museum of Natural Science Taichung Taiwan; 5 Department of Biological Sciences, National Sun Yat-sen University, No. 70 Lienhai Rd., Gushan District, Kaohsiung City 80424, Taiwan National Sun Yat-sen University Kaohsiung Taiwan

**Keywords:** Distribution, diversity, Easter egg weevil, host plant, *Pachyrrhynchus*, synonym, type

## Abstract

The genus *Pachyrhynchus* comprises a group of entimine weevils with diverse body coloration, endemic to the islands of Southeast Asia, with the highest diversity of species found in the Philippines. In the past ten years, many new species have been discovered, necessitating an updated list of *Pachyrhynchus* weevils for future study. This checklist provides the valid names, synonyms, type depository, type locality, and distribution of each *Pachyrhynchus* species, with ecological information such as host plants and life history. Species are further classified into defined species groups based on recent revisions using morphology and genetic analyses. A region-based analysis across eight major faunal areas highlights spatial patterns in species richness. Since the first *Pachyrhynchus* species was described 200 years ago, a total of 179 species and 43 subspecies (excluding nominotypical subspecies) have been recognized, with 45.8% of *Pachyrhynchus* species distributed in the Greater Luzon Faunal Region and 39.1% in Greater Mindanao. However, most species still lack ecological information, indicating the difficulty in collecting these data, and further investigations are needed.

## ﻿Introduction

The genus *Pachyrhynchus* Germar, 1824 (Curculionidae, Entiminae, Pachyrhynchini) (Alonso-Zarazaga and Lyal 1999) is a group of insular broad-nosed weevils distributed from Indonesia to the Ishigaki and Iriomote islands of Japan, including the Philippines, and the Lanyu (Orchid Island/Botel Tobago) and Ludao (Green Island) islands in southeastern Taiwan. They are a group of flightless beetles characterized by completely fused elytra and a wide variety of elytral coloration, with the majority of species found in the Philippines.

**Figure 1.**
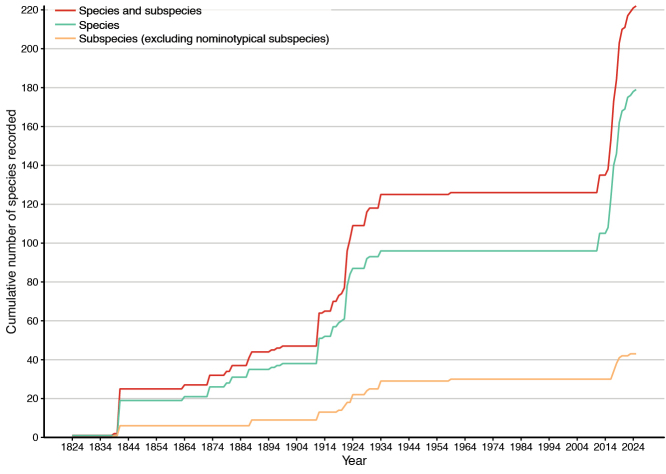
Cumulative number of species published since 1824.

The genus *Pachyrhynchus* was established by Germar (1824) based on a specimen of *P.moniliferus* collected from Manila, Luzon Island (Philippines). Members of this genus are characterized by a prominent short and thick rostrum, short antennae, absence of scutellar shield, and fused elytra, resulting in flightlessness. Four researchers, George Robert Waterhouse (United Kingdom), Louis Alexandre Auguste Chevrolat (French), Wilhelm Schultze (Germany), and Karl Maria Heller (Austria), were the most influential authorities in the development of *Pachyrhynchus* taxonomy. Since the first species was described, Waterhouse and Chevrolat contributed more than 20 species in 1841. Later, Heller and Schultze described more than 70 species during 1899–1934, bringing the total to over 120 species by 1934 (Fig. 1). Heller (1912) published the first monograph on *Pachyrhynchus*, and categorized *Pachyrhynchus* weevils into seven groups. Later, Schultze (1923, 1924) expanded the classification into 15 groups both primarily based on coloration patterns, and provided keys to the species within these groups.

The taxonomy of *Pachyrhynchus* remained poorly studied until 80 years later (Yoshitake 2012). Since 2012, approximately 90 new synonyms and combinations have been proposed, mostly by Hiraku Yoshitake (see Yoshitake 2017a, 2019a, b, 2020; Yoshitake et al. 2019a), Maurizio Bollino (see Bollino et al. 2017), Anita Rukmane-Bārbale and Analyn Anzano Cabras (see Rukmane and Barševskis 2016; Rukmane-Bārbale 2020a). These taxonomic works have enriched knowledge on the diversity of *Pachyrhynchus*, resulting in 179 described species and 43 subspecies to date. Furthermore, six new species groups have been proposed: the *absurdus* species group (Rukmane 2017), the *amabilis* species group (Bollino et al. 2017; Bollino et al. 2020), the *atrocyaneus* species group (Bollino 2022), the *pinorum* species group (Rukmane-Bārbale 2020b), the *speciosus* species group (Rukmane 2019a), and the *schoenherri* species group (Bollino et al. 2017). These studies used genitalia and the endophallus of the aedeagus as reliable diagnostic characters.

Rukmane (2018a) published a checklist of *Pachyrhynchus* based on an extensive review of existing literature, and proposed a total of 40 new synonyms. Recently, *Pachyrhynchus* researchers have not only focused on taxonomic re-evaluation but have also paid more attention to various other topics, including ecology (Hsu et al. 2017; Tseng et al. 2021), color signaling (Tseng et al. 2014; Lee et al. 2018; Hsu et al. 2021), dispersal mechanisms (Ye et al. 2018; Lin et al. 2021), and phylogenetic studies (Tseng et al. 2018; Ye et al. 2022; Van Dam et al. 2023; Chen et al. 2025). Considering the above, new studies and integrated literature reviews have enhanced our understanding of the taxonomy and distribution of *Pachyrhynchus* species. This study aims to present an updated species checklist of the genus *Pachyrhynchus*, with synonyms, type localities, depositories, distributions, host plant information, and species group classifications from various approaches.

## ﻿Materials and methods

### ﻿Checklist and literature review

The valid names, synonyms, type depositories, type localities, distributions, and species groups of each *Pachyrhynchus* species are provided. Verification of type depositories is primarily based on the original descriptions, museum collection lists, or personal visits to the depositories. If the type locality cannot be confirmed because the identity of the type specimens (whether they are nominal subspecies or different subspecies) is unverified in the museum collection list; an asterisk (*) is used to indicate uncertainty regarding the depository. Distribution data were obtained by reviewing the literature. Additional information on host plant species, as reported in the literature (citations listed below) or observed by the authors (noted as “This study”) in the wild, is also provided.

The materials we examined are deposited in the following institutions:

**NHMUK**Natural History Museum, London, United Kingdom

**SDEI**Senckenberg Deutsches Entomologisches Institut, Müncheberg, Germany

Other materials from the literature or from museum collection lists are also cited, which are deposited in the following institutions and personal collections:

**CFS** Franco Sandel private collection, Miane, Italy

**CMU-MZ** Central Mindanao University, University Museum, Zoological Section, Philippines

**DHA** Derek Helmericks private collection, Anchorage, Alaska, USA

**DUBC** Daugavpils University, Institute of Life Sciences and Technology, Coleopterological Research Centre, Ilgas, Daugavpils District, Latvia

**HUMS**Hokkaido University Museum, Sapporo, Japan

**MBLI** Maurizio Bollino private collection, Lecce, Italy

**MCKUM**Munetoshi Maruyama Collection at the Kyushu University Museum, Fukuoka, Japan

**MNHN**National Museum of Natural History, Paris, France

**MZZC** Ming-Zhi Zhao private collection, Zhuhai, China

**NHRS**Naturhistoriska riksmuseet, Stockholm, Sweden

**NIAES**Institute for Agro-Environmental Sciences, NARO, Tsukuba, Japan

**PCKS** Private collection of Kaoru Sakai, Tokyo, Japan

**PNMNH** Philippine National Museum of Natural History, Manila, Philippines

**SMTD** Senckenberg Naturhistorische Sammlungen, Museum für Tierkunde, Dresden, Germany

**UMCRC** University of Mindanao Coleoptera Research Center, Davao City, Philippines

**UPLB**University of the Philippines Los Baños, Los Baños, Philippines

**ZMHB** Naturkundemuseum Berlin (former Zoologische Museum der Humboldt-Universität), Berlin, Germany

### ﻿Distributional analysis

**Figure 2.**
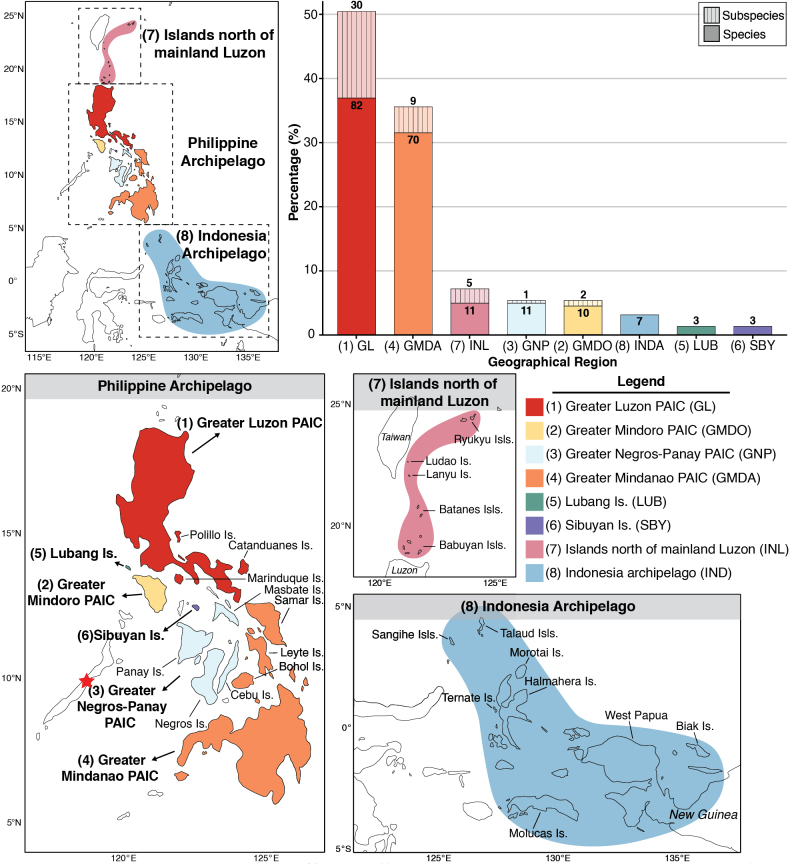
Species distribution of *Pachyrhynchus* weevils. Different colors on the map represent the defined regions. The blank islands of the Philippine archipelago indicate areas that do not belong to any of the four PAICs. Some Pachyrhynchini but no *Pachyrhynchus* species are found in the Palawan region, marked with a red star. The map was generated using the ggplot2 package in R (Wickham 2016). The bar plot shows the percentage of species distribution across different regions. The numbers in the bars represent the number of species or subspecies (excluding nominotypical subspecies).

Based on geographical distribution of islands, we divided the species distribution into eight regions, including the four Pleistocene Aggregate Island Complexes (PAICs) faunal regions (Heaney 1986; Brown and Diesmos 2002; Brown et al. 2013), Indonesian archipelago, islands north of mainland Luzon, Lubang Island and Sibuyan Island (Fig. 2). These regions are defined as follows:

1. Greater Luzon PAIC: Luzon Island, Polillo Island, Catanduanes Island, Marinduque Island
2. Greater Mindoro PAIC: Mindoro Island
3. Greater Negros-Panay PAIC: Panay Island, Negros Island, Cebu Island, Masbate Island
4. Greater Mindanao PAIC: Samar Island, Leyte Island, Bohol Island, Biliran Island, Dinagat Island, Siargao Island, Basilan Island, Bucas Grande Island
5. Lubang Island
6. Sibuyan Island
7. Islands north of mainland Luzon: Ishigaki Island, Iriomote Island, Lanyu Island, Ludao Island, Batanes Islands, Babuyan Islands
8. Indonesian archipelago: Maluku Islands and West Papua Province with its adjacent islands of Indonesian Papua

## ﻿Results

The genus *Pachyrhynchus* currently comprises 179 described species and 43 subspecies. The results revealed that *Pachyrhynchus* is distributed in Japan (1 species, 0.6%), Taiwan (6 species, 3.4%), the Philippines (168 species, 93.9%), and Indonesia (7 species, 3.9%).

**Order Coleoptera Linnaeus**

**Suborder Polyphaga Emery**

**Infraorder Cucujiformia Lameere**

**Superfamily Curculionoidea Latreille**

**Family Curculionidae Latreille**

**Subfamily Entiminae Schöenherr**

**Tribe Pachyrhynchini Schöenherr**

**Genus *Pachyrhynchus* Germar, 1824**

= *Sphaerogaster* Dejean, 1821

= *Somatodes* Schoenberr, 1823

= *Sphoerogaster* Latreille, 1825

= *Sphaerogaster* Sturm, 1826

= *Sphoenogaster* Bertbold, 1827

= *Pachirhinus* Latreille, 1828

= *Pochyrhynchus* Laporte, [1840]

= *Pachyrhinchus* Desmarest, [1842]

= *Pachyrhincus* Desmarest, [1842]

= *Pachyrrhynchus* Gemminger & Harold, 1871

***Pachyrhynchusamabilis* Schultze, 1922**

**Type depository.**SMTD (holotype).

**Type locality.** Philippines (Mindanao Is.: Bukidnon Prov., Lindaban).

**Distribution.** Philippines: Mindanao Is. (Bukidnon, Lindaban, Intavas; Zamboanga; Davao City; Lanao; Cotabato; Mt. Kalatungan) (Schultze 1922a, 1924; Rukmane 2019b; Rukmane-Bārbale and Cabras 2021; Cabras et al. 2022a).

**Remarks.** Two individuals were collected from the Bracken fern, *Pteridiumaquilinum* (Dennstaedtiaceae) in Davao City (Cabras et al. 2022a), suggesting it as a potential host plant.

***Pachyrhynchusanellifer* Heller, 1912**

*Pachyrhynchusannulatus* Behrens, 1887: 256. Synonymized by Schultze (1924: 318).

**Type depository.**SMTD (holotype).

**Type locality.** Philippines (Luzon Is.: Benguet Prov., Irisan River).

**Distribution.** Philippines: Luzon Is. (Benguet, Baguio, Mt. Santo Tomas; Ifugao; Nueva Vizcaya) (Schultze 1924; Rukmane 2019b, c; Rukmane-Bārbale and Cabras 2021).

**Remarks.** The conspecificity of *P.annulatus* Behrens, 1887 and *P.anellifer* Heller, 1912 was recognized by Schultze (1924). Although the former name has priority, it is preoccupied by *P.annulatus* Chevrolat, 1881, rendering it permanently invalid. Consequently, the valid name of the species is *P.anellifer* Heller, 1912.

***Pachyrhynchusanichtchenkoi* Rukmane & Barševskis, 2016**

**Type depository.**DUBC (holotype, paratypes).

**Type locality.** Philippines (Mindanao Is: Bukidnon Prov., Mt. Kalatungan).

**Distribution.** Philippines: Mindanao Is. (Agusan; Bukidnon, Mt. Kalatungan, Intavas, Cabanglasan, Mt. Kiamo; Davao de Oro; Compostela; Lanao del Sur; Sarrangani, Kiamba; Zamboanga) (Cabras et al. 2017; Rukmane 2019b; Rukmane-Bārbale and Cabras 2021).

***Pachyrhynchusannulatus* Chevrolat, 1881**

**Type depository.** Unknown.

**Type locality.** Philippines (Chevrolat 1881a).

**Distribution.** Philippines: Luzon Is. (Benguet, Mt. Pulog; Ifugao; Mountain Prov.) (Schultze 1924; Rukmane 2019b).

**Remarks.***Pachyrhynchusannulatus* Behrens, 1887 is a junior primary homonym of *P.annulatus* Chevrolat, 1881; therefore, it is permanently invalid.

***Pachyrhynchusantonkozlovi* Rukmane & Barševskis, 2016**

**Type depository.**DUBC (holotype, paratypes).

**Type locality.** Philippines (Mindanao Is.: Sarangani province, Malungon).

**Distribution.** Only known from the type locality.

***Pachyrhynchusapicatus* Schultze, 1922**

**Type depository.**SMTD (syntypes).

**Type locality.** Philippines (Polillo Is.).

**Distribution.** Philippines: Luzon Is. (Aurora, Dingalan, Sierra Madre); Marinduque Is.; Mindoro Is. (Mt. Halcon); Polillo Is. (Schultze 1922b; Schultze 1924; Háva and Rukmane 2018; Rukmane 2019b, 2019c; Rukmane-Bārbale and Cabras 2021).

***Pachyrhynchusapocyrtoides* Schultze, 1922**

**Type depository.**SMTD (holotype).

**Type locality.** Philippines (Mindanao Is.: Bukidnon Prov., Lindaban).

**Distribution.** Philippines: Mindanao Is. (Agusan; Bukidnon; Cotabato; Davao del Sur; Surigao del Norte) (Schultze 1922a; Schultze 1923; Rukmane-Bārbale and Cabras 2021).

***Pachyrhynchusapoensis* Yoshitake, 2012**

*Pachyrhynchuspseudapoensis* Rukmane & Barševskis, 2016: 94. Synonymized by Yoshitake (2016a: 197).

**Type depository.**NIAES (holotype, paratypes); PCKS (paratypes).

**Type locality.** Philippines (Mindanao Is.: Mt. Apo).

**Distribution.** Philippines: Mindanao Is. (Bukidnon; Cotabato; Davao del Sur, Mt. Apo; Sarrangani) (Cabras and Yoshitake 2016; Rukmane-Bārbale and Cabras 2021; Cabras et al. 2022a).

**Host plants.***Impatiensplatypetala* (Balsaminaceae), *Melastoma* sp. (Melastomaceae), *Diplaziumdavaoense* (Woodsiaceae) and *Saurauia* sp. (Actinidiaceae) (Cabras and Yoshitake 2016)

**Remarks.***Pachyrhynchusapoensis* belongs to the *P.schoenherri* species group and conforms to the original described characters and distribution to the *P.schoenherri* species group (Bollino et al. 2017). Rukmane and Barševskis (2016) described *P.pseudapoensis*, based on a single female specimen from Luzon, which was treated as a synonym as *P.apoensis* by Yoshitake (2016a). The locality “North Luzon” may need to be reevaluated, as *P.apoensis* and its related species are known only from Mindanao and its adjacent islands (Yoshitake 2016a).

***Pachyrhynchusardentius* Schultze, 1919**

**Type depository.**SMTD (holotype).

**Type locality.** Philippines (Siargao Is.).

**Distribution.** Philippines: Siargao Is.; Mindanao Is. (Bukidnon, Cotabato, Davao del sur, Sarangani; Mt. Apo) (Schultze 1924; Rukmane 2019b; Rukmane-Bārbale and Cabras 2021).

***Pachyrhynchusargus* Pascoe, 1873**

**Type depository.**NHMUK (holotype).

**Type locality.** Philippines.

**Distribution.** Philippines: Luzon Is. (Benguet, Baguio, Mt. Santo Tomas, Trinidad, Mt. Trail, Pauai, Mt. Polis, Mt. Pulog; Manila) (Schultze 1924; Rukmane 2018a, 2019b, c).

***Pachyrhynchusatrocyaneus* Schultze, 1922**

**Type depository.**SMTD (syntypes).

**Type locality.** Mindanao (Zamboanga Prov.: Zamboanga).

**Distribution.** Philippines: Mindanao Is. (Zamboanga) (Schultze 1922a; Schultze 1924; Rukmane-Bārbale and Cabras 2021).

***Pachyrhynchusatronitens* Yoshitake, 2019**

**Type depository.**NIAES (holotype, paratypes); KUM (paratypes).

**Type locality.** Philippines (Luzon Is.: Kalinga Prov., Pinukpuk).

**Distribution.** Philippines: Luzon Is. (Abra; Kalinga) (Yoshitake 2019c; Rukmane-Bārbale and Cabras 2021).

***Pachyrhynchusbaluganus* Schultze, 1924**

**Type depository.**SMTD (holotype).

**Type locality.** Philippines (Luzon Is.: Rizal Prov., Mount San Isidro).

**Distribution.** Philippines: Luzon Is. (Quirino; Rizal) (Rukmane-Bārbale and Cabras 2021).

***Pachyrhynchusbanglas* Bollino, Sandel & Rukmane, 2017**

**Type depository.**SMTD (holotype); MBLI, DUBC, NIAES, KUM, CFS (paratypes).

**Type locality.** Philippines (Mindanao Is.: Bukidnon Prov., Kabanglasan).

**Distribution.** Philippines: Mindanao Is. (Bukidnon, Kabanglasan, Cabanglasan, San Fernando, Intavas, Malaybalay; Summit Mountain Prov.) (Bollino et al. 2017).

***Pachyrhynchusbarsevskisi* Rukmane, 2016**

**Type depository.**DUBC (holotype, paratypes).

**Type locality.** Philippines (Luzon Is.: Aurora Prov., Dingalan).

**Distribution.** Philippines: Luzon Is. (Aurora, Dingalan, Ditumabao, Casiguran; Sierra Madre; Nueva Ecija, Caranglan; Quirino, Madela; Nueva Vizcaya, Alfonso Castañeda, Belance) (Rukmane-Bārbale 2020b; Rukmane-Bārbale and Cabras 2021).

***Pachyrhynchusbasilanus* Heller, 1923**

**Type depository.**SMTD (holotype).

**Type locality.** Philippines (Basilan Is.).

**Distribution.** Philippines: Basilan Is.; Mindanao Is. (Zamboanga City, Labuan) (Heller 1923a; Bollino 2022).

***Pachyrhynchusbenguetanus* Schultze, 1924**

**Type depository.**SMTD (syntypes).

**Type locality.** Philippines (Luzon Is.: Benguet Prov.).

**Distribution.** Philippines: Luzon Is. (Benguet, Ifugao) (Rukmane-Bārbale and Cabras 2021).

***Pachyrhynchusbollinoi* Rukmane-Bārbale, 2020**

**Type depository.**DUBC (holotype, paratypes).

**Type locality.** Philippines (Luzon Is.: Mountain Prov., Barlig).

**Distribution.** Philippines: Luzon Is. (Ifugao, Banaue; Mountain Prov., Barlig; Nueva Vizcaya, Kayapa) (Rukmane-Bārbale 2020c).

***Pachyrhynchusbucasanusbucasanus* Schultze, 1922**

**Type depository.**SMTD (holotype).

**Type locality.** Philippines (Bucas Grande Is.: Surigao del Norte Prov.).

**Distribution.** Only known from the type locality (Schultze 1922a).

***Pachyrhynchusbucasanusornatus* Schultze, 1934**

**Type depository.**SMTD (syntypes).

**Type locality.** Philippines (Samar Is.: Borongan).

**Distribution.** Only known from the type locality.

***Pachyrhynchuscabrasae* Rukmane & Barševskis, 2016**

**Type depository.**DUBC (holotype, paratypes).

**Type locality.** Philippines (Mindanao Is.: Bukidnon Prov., Mt. Kalatungan).

**Distribution.** Philippines: Mindanao Is. (Agusan; Bukidnon, Mt.

Kalatungan, Cabanglasan, Mt. Kiamo; Davao del Sur) (Cabras et al. 2017; Rukmane-Bārbale and Cabras 2021).

***Pachyrhynchuscaeruleovittatus* Yoshitake, 2012**

**Type depository.**NIAES (holotype, paratypes).

**Type locality.** Philippines (Mindanao Is.: Mt. Parker).

**Distribution.** Philippines: Mindanao Is. (Sarangani, South Cotabato)

(Rukmane-Bārbale and Cabras 2021).

***Pachyrhynchuscaeruleus* Yoshitake, 2019**

**Type depository.** KUM (holotype, paratypes).

**Type locality.** Philippines (Luzon Is.: Aurora Prov., central part of Sierra Madre Mountain Range).

**Distribution.** Only known from the type locality (Yoshitake 2019d).

***Pachyrhynchuscagayanus* Heller, 1929**

**Type depository.**SMTD (holotype).

**Type locality.** Philippines (Luzon Is.: Cagayan Prov., Tuao).

**Distribution.** Philippines: Luzon Is. (Aurora, Dingalan; Cagayan; Isabela; Kalinga; Quirino) (Rukmane-Bārbale and Cabras 2021).

***Pachyrhynchuscallainimaculatus* Yoshitake, 2017**

**Type depository.**NIAES (holotype).

**Type locality.** Philippines (Luzon Is.: Cagayan Prov., Sierra Madre).

**Distribution.** Only known from the type locality (Yoshitake 2017b).

***Pachyrhynchuscebrem* Patano & Rukmane-Bārbale, 2022**

**Type depository.**CMU-MZ (holotype, paratypes).

**Type locality.** Philippines (Mindanao Is.: Davao de Oro Prov., Maragusan, Mt. Candalaga).

**Distribution.** Only known from the type locality.

***Pachyrhynchuschamissoi* Schultze, 1922**

**Type depository.**SMTD (syntypes).

**Type locality.** Philippines (Mindanao Is.: Bukidnon Prov., Lindaban).

**Distribution.** Philippines: Mindanao Is. (Bukidnon, Lindabon); Pollilo Is. (Schultze 1922a; Schultze 1924; Rukmane 2019c).

***Pachyrhynchuschlorites* Chevrolat, 1881**

*Pachyrhynchusrutilans* Behrens, 1887: 247. Synonymized by Schultze (1924: 327).

**Type depository.**NHRS (syntypes).

**Type locality.** Philippines (Chevrolat 1881b).

**Distribution.** Philippines: Babuyan Is.; Calayan Is.; Luzon Is. (Aurora; Cagayan; Ifugao, Banaue; Ilocos Norte; Isabela; Nueva Vizcaya; Quirino; Manila) (Schultze 1924; Rukmane 2019b; Rukmane-Bārbale and Cabras 2021).

***Pachyrhynchuschloritesinsularis* Kano, 1929**

**Type depository.** Unknown.

**Type locality.** Taiwan (Taitung County, Lanyu Is. (= Kotosho)).

**Distribution.** Only known from the type locality.

**Host plants.** According to our field observations, they feed on *Bischofiajavanica* (Phyllanthaceae) in Lanyu (this study).

**Remarks.***P.insularis* Kano, 1929, from Lanyu was originally considered a distinct species. However, Schultze (1937), after examining specimens of *P.chlorites* from Calayan with Kano’s description, regarded the Lanyu population as a variety of *P.chlorites*. According to the ICZN (Article 45.6.4.), a variety proposed before 1961 is automatically treated as subspecies. Whether *P.chloritesinsularis* in Lanyu is a distinct species requires further clarification.

***Pachyrhynchuschrysocyaneus* Rukmane-Bārbale, 2024**

**Type depository.**DUBC (holotype, paratypes).

**Type locality.** Philippines (Luzon Is.: Nueva Ecija Prov., Gabaldon).

**Distribution.** Only known from the type locality.

***Pachyrhynchuschrysomaculatus* Bollino, 2022**

**Type depository.**SMTD (holotype); MBLI, MCKUM, CFS (paratypes).

**Type locality.** Philippines (Mindanao Is.: Bukidnon Prov., Cabanglasan).

**Distribution.** Only known from the type locality.

***Pachyrhynchuscinereomaculatus* Rukmane-Bārbale, 2020**

**Type depository.**DUBC (holotype, paratypes).

**Type locality.** Philippines (Luzon Is.: Ifugao Prov., Banaue).

**Distribution.** Philippines: Luzon Is. (Aurora; Ifugao; Nueva Vizcaya, Kasibu) (Rukmane-Bārbale 2020c).

***Pachyrhynchuscingulatus* Pascoe, 1873**

**Type depository.** Unknown.

**Type locality.** Indonesia (Morotai Is.).

**Distribution.** Only known from the type locality.

**Remarks.** The type specimen named by Pascoe might be deposited in NHMUK. However, JFT did not find it in the collections.

***Pachyrhynchuscirculatus* Heller, 1912**

**Type depository.**NHMUK (syntype), SMTD (syntype).

**Type locality.** Philippines (Catanduanes Is.).

**Distribution.** Philippines: Catanduanes Is. (Schultze 1923; Rukmane 2019b).

***Pachyrhynchuscirculimaculatus* Yoshitake, 2019**

**Type depository.**NIAES (holotype).

**Type locality.** Philippines (Mindanao Is.: Misamis Occidental Prov.).

**Distribution.** Philippines: Mindanao Is. (Agusan del Norte, Misamis Occidental) (Yoshitake 2019e).

***Pachyrhynchusconformis* Yoshitake, 2017**

**Type depository.**NIAES (holotype, paratypes); CFS, MBLI (paratypes).

**Type locality.** Philippines (Samar Is.: Samar Prov.).

**Distribution.** Philippines: Samar Is. (Samar, Hinabangan) (Yoshitake 2017c, Rukmane-Bārbale and Cabras 2021).

***Pachyrhynchusconfusus* Schultze, 1923**

**Type depository.**NHMUK (syntypes), SMTD (syntypes).

**Type locality.** Philippines (Luzon Is.: Los Baños).

**Distribution.** Philippines: Luzon Is. (Benguet, Mt. Makiling; Laguna, Los Baños) (Rukmane 2019c)

**Host plants.***Acrostichumaureum* (Pteridaceae) (Schultze 1924).

***Pachyrhynchuscongestuscongestus* Pascoe, 1873**

*Pachyrhynchusluteoguttatus* Chevrolat, 1881b: 360. Synonymized by Schultze (1924: 324).

**Type depository.**NHMUK (holotype).

**Type locality.** Philippines.

**Distribution.** Philippines: Luzon Is. (Aurora; Benguet, Baguio, Atoc, Mt. Santo Tomas, Mt. Trail, P.I. Atok, Mt. Pawai, Mt. Mirador, Trinidad; Manila; Mountain Prov., Bontoc; Nueva Vizcaya, Kasibu; Ifugao; Quirino; Kallinga) (Schultze 1924; Rukmane 2019b, 2019c; Rukmane-Bārbale and Cabras 2021).

***Pachyrhynchuscongestusaedamlayroni* Rukmane, 2019**

**Type depository.**DUBC (holotype, paratypes).

**Type locality.** Philippines (Luzon Is.: Ilocos Norte Prov., Adams).

**Distribution.** Only known from the type locality.

***Pachyrhynchuscongestuscoerulans* Kraatz, 1888**

**Type depository.**SDEI (*).

**Type locality.** Philippines.

**Distribution.** Philippines: Luzon Is. (Mountain Prov., Bontoc, Mt. Polis; Kalinga) (Schultze 1924; Rukmane 2019c).

***Pachyrhynchuscongestusimmarginatus* Kraatz, 1888**

**Type depository.**SDEI (*).

**Type locality.** Philippines.

**Distribution.** Philippines: Luzon Is. (Benguet, Mt. Data) (Schultze 1924).

***Pachyrhynchuscongestusmirabilis* Yoshitake, 2017**

**Type depository.**NIAES (holotype, paratypes).

**Type locality.** Philippines (Luzon Is.: Nueva Vizcaya Prov., Lohong).

**Distribution.** Philippines: Luzon Is. (Cagayan Vally region; Nueva Vizcaya, Belance, Lohong) (Yoshitake 2017b).

***Pachyrhynchuscongestusocellatus* Schultze, 1924**

**Type depository.**SMTD (*).

**Type locality.** Philippines (Luzon Is.: Benguet Prov., settlement Bokod near Mount Pandan).

**Distribution.** Only known from the type locality.

***Pachyrhynchuscongestuspavonius* Heller, 1921**

**Type depository.**SMTD (*).

**Type locality.** Philippines (Luzon Is., Nueva Vizcaya Prov., Imugan).

**Distribution.** Philippines: Luzon Is. (Nueva Vizcaya, Imugan) (Schultze 1924).

***Pachyrhynchusconsobrinus* Schultze, 1922**

**Type depository.**SMTD (syntypes).

**Type locality.** Philippines (Luzon Is., Mountain Prov., Bontoc).

**Distribution.** Philippines: Luzon Is. (Abra, Malibcong, Tineg; Apayao, Conner; Benguet; Moutain Prov., Barlig, Bontoc, Mt. Polis; Kalinga, Pinukpuk) (Schultze 1922b; Schultze 1924; Rukmane-Bārbale 2020b; Rukmane-Bārbale and Cabras 2021).

***Pachyrhynchuscorpulentus* Schultze, 1922**

**Type depository.**SMTD (syntypes).

**Type locality.** Philippines (Mindanao Is.: Bukidnon Prov., Lindabon).

**Distribution.** Philippines: Mindanao Is. (Bukidnon, Lindabon; Zamboanga) (Schultze 1922a; Schultze 1924; Rukmane 2019b).

***Pachyrhynchuscorpulentusbalatukan* Patano & Macalaba, 2023**

**Type depository.**CMU-MZ (holotype, paratypes).

**Type locality.** Philippines (Mindanao Is.: Misamis Oriental Prov., Gingoog City, Mt. Balatukan).

**Distribution.** Only known from the type locality.

***Pachyrhynchuscroesus* Oberthur, 1879**

**Type depository.**NHRS (holotype).

**Type locality.** Indonesia (Sangir).

**Distribution.** Indonesia: Sangihe Isls. (Sangir Is.); Talaud Isls. (Kabaruan Is., Salibabu Is.) (Schultze 1924; Yoshitake et al. 2016; Yoshitake 2018a; Rukmane 2018b, 2019b).

***Pachyrhynchuscruciatus* Schultze, 1923**

**Type depository.**SMTD (*).

**Type locality.** Philippines (Luzon Is.: Tayabas Prov., Baler).

**Distribution.** Philippines: Luzon Is. (Abra; Aurora; Ifugao, Banaue; Sierra Madre; Dingalon; Manila) (Rukmane and Barševskis 2016; Rukmane 2018c; Rukmane-Bārbale and Cabras 2021).

***Pachyrhynchuscumingiicumingii* Waterhouse, 1841**

**Type depository.**SMTD (*).

**Type locality.** Philippines.

**Distribution.** Philippines: Leyte Is. (Leyte); Mindanao Is. (Bukidnon, Mt. Kiamo, Cabanglasan, Intavas, Mt. Kalatungan, Panamokan; Sarangani, Kiamba); Bohol Is.; Samar Is. (Hinabangan, Marabut) (Waterhouse 1841; Cabras et al. 2017).

**Remarks.** The original spelling of this species is “cumingii,” with a double “i,” following the ICZN (Articles 31.1.1 and 31.1.2); however, many references in the literature have incorrectly used the spelling “cumingi”.

***Pachyrhynchuscumingiiboholensis* Schultze, 1924**

**Type depository.**SMTD (*).

**Type locality.** Philippines (Bohol Is.: Bilar).

**Distribution.** Only known from the type locality.

***Pachyrhynchusdavaoensis* Schultze, 1934**

**Type depository.**SMTD (holotype).

**Type locality.** Philippines (Mindanao Is.: Davao Prov., Apo Volcano).

**Distribution.** Philippines: Mindanao Is. (Bukidnon, Cabanglasan; Cotabato, Tboli; Surigao del Sur, Esperanza, San Miguel) (Rukmane 2019a; Rukmane-Bārbale and Cabras 2021; Cabras et al. 2022a).

***Pachyrhynchusdecussatusdecussatus* Waterhouse, 1841**

**Type depository.**NHMUK (holotype).

**Type locality.** Philippines.

**Distribution.** Philippines: Catanduanes Is. (Virac) (Waterhouse 1841; Schultze 1923); Luzon Is. (south Luzon, Mt. Bulusan) (Rukmane-Bārbale 2020d).

***Pachyrhynchusdecussatuscatanduanensis* Rukmane-Bārbale, 2020**

**Type depository.**DUBC (holotype, paratype).

**Type locality.** Philippines (Catanduanes Is.: Catanduanes Prov., Pandan).

**Distribution.** Only known from the type locality.

***Pachyrhynchusdisargus* Rukmane, 2019**

**Type depository.**DUBC (holotype, paratypes).

**Type locality.** Philippines (Luzon Is.: Nueva Vizcaya Prov., Kasibu).

**Distribution.** Philippines: Luzon Is. (Nueva Vizcaya, Kasibu, Kayapa) (Rukmane 2019d).

***Pachyrhynchusdisgestus* Heller, 1929**

**Type depository.**SMTD (holotype).

**Type locality.** Philippines (Luzon Is.: Nueva Vizcaya Prov., Bayombong).

**Distribution.** Philippines: Luzon Is. (Ifugao, Moutain Prov., Nueva Vizcaya) (Rukmane 2019b; Rukmane-Bārbale and Cabras 2021).

***Pachyrhynchusdohrni* Behrens, 1887**

**Type depository.** Unknown.

**Type locality.** Philippines.

**Distribution.** Philippines: Luzon Is. (Manila) (Rukmane 2019b).

***Pachyrhynchusdomino* Rukmane, 2016**

**Type depository.**DUBC (holotype).

**Type locality.** Philippines (Mindoro Is.: Mindoro Oriental Prov., Mt. Halcon).

**Distribution.** Philippines: Mindoro Is. (Mindoro Oriental Prov., Mt. Halcon, Baco) (Rukmane and Háva 2020; Rukmane-Bārbale and Cabras 2021).

***Pachyrhynchusdubiosus* Schultze, 1922**

**Type depository.**SMTD (syntypes).

**Type locality.** Philippines (Luzon Is.: Benguet Prov., Berg Santo Tomas und Haight’s Place).

**Distribution.** Philippines: Luzon Is. (Benguet, Loo, Mt. Santo Tomas, Pauai; Mountain Prov.) (Schultze 1922b; Schultze 1924; Rukmane 2019b, 2019c).

***Pachyrhynchuselegans* Waterhouse, 1842**

*Pachyrhynchuseos* Heller, 1924: 171. Synonymized by Bollino, Sandel and Rukmane (2017: 201).

**Type depository.**NHMUK (holotype).

**Type locality.** Unknown.

**Distribution.** Philippines: Samar Is. (Northern Samar, Lope De Vega; Samar, Hinabangan) (Waterhouse 1842; Rukmane-Bārbale and Cabras 2021).

***Pachyrhynchuseques* Heller, 1912**

**Type depository.**SMTD (holotype).

**Type locality.** Philippines (Luzon Is.: Cagayan, Abulog River).

**Distribution.** Philippines: Luzon Is. (Cagayan, Abulog River) (Schultze 1924).

***Pachyrhynchusequester* Heller, 1929**

**Type depository.**SMTD (holotype).

**Type locality.** Philippines (Luzon Is.: Benguet Prov., Baguio).

**Distribution.** Only known from the type locality.

***Pachyrhynchuserichsonierichsoni* Waterhouse, 1841**

Pachyrhynchuserichsonivar.chrysocompsus Heller, 1912: 307. Synonymized by Schultze (1923: 663).

**Type depository.**NHMUK (syntype); SMTD (syntype).

**Type locality.** Philippines.

**Distribution.** Philippines: Dinagat Is.; Leyte Is. (Sogod; Cabalian); Samar Is. (Hinabanga); Luzon Is. (Sorsogon; Cagayan; Ifuga, Banaue; Sierra Madre; Aurora, Dingalan, Labulo; Nueva Vizcaya, Belance; Quirino, Disimungal, Madela); Marinduque Is. (Boac; Buenavista); Mindoro Is. (Baco); Mindanao Is. (Bukidnon, Mt. Kiamo, Cabanglasan, Intavas, Panamokan; Cotobato, Kidapawan, Mt. Apo; Surigao del Sur, Esperanza; Davao City, Marilog District, Catigan, Toril, Tamayong, Calinan) (Waterhouse 1841; Schultze 1923; Cabras et al. 2017; Rukmane 2019b; Rukmane-Bārbale and Cabras 2021; Cabras et al. 2022a).

**Host plants.** The *P.erichsoni* complex has been documented on the following plants: *Melastomamalabathricum* (Melastomataceae), *Lithocarpusboholensis* (Fagaceae), *Dendrocnide* sp. (Urticaceae), *Callicarpa* sp. (Lamiaceae), *Philodendron* sp. (Araceae), and *Amaranthus* sp. (Amaranthaceae) (Cabras et al. 2022a).

***Pachyrhynchuserichsonieschscholtzii* Waterhouse, 1841**

**Type depository.**NHMUK (syntype); SMTD (syntype).

**Type locality.** Philippines.

**Distribution.** Philippines: Luzon Is. (Laguna Prov., Paete, Mt. Banahao; Ilocos Norte Prov., Bangui); Polillo Is.; Marinduque Is. (Waterhouse 1841; Schultze 1923, 1924; Rukmane-Bārbale and Cabras 2021).

***Pachyrhynchuserosus* Schultze, 1920**

**Type depository.**SMTD (syntypes).

**Type locality.** Philippines (Luzon Is.: Benguet Prov., mountain trail near Atoc).

**Distribution.** Philippines: Luzon Is. (Benguet, Abatan, La Trinidad) (Rukmane-Bārbale and Cabras 2021).

***Pachyrhynchusesperanza* Bollino, Sandel & Rukmane, 2017**

**Type depository.**SMTD (holotype); MBLI, DUBC, NIAES, KUM, CFS (paratypes).

**Type locality.** Philippines (Mindanao Is.: Agusan del Sur Prov.).

**Distribution.** Philippines: Mindanao Is. (Agusan del Sur, Esperanza, Sibagat, Talacogon; Caraga region; Bukidnon, Cabanglasan, Valencia; Surigao del Sur).

***Pachyrhynchusfaisali* Bollino, 2023**

**Type depository.**SMTD (holotype).

**Type locality.** Indonesia (New Guinea Is.: West Papua, South Sorong Regency, Teminabuan).

**Distribution.** Only known from the type locality.

***Pachyrhynchusfelipeae* Rukmane & Cabras, 2018**

**Type depository.**DUBC (holotype, paratypes).

**Type locality.** Philippines (Panay Is.: Antique Prov., Culasi, Aklan).

**Distribution.** Philippines: Panay Is. (Antique, Culasi, Aklan).

**Remarks.** This species is very similar and sympatric with *P.franciscoi*Rukmane and Cabras (2018b) from Panay Island, Philippines.

***Pachyrhynchusflorulentus* Yoshitake, 2019**

**Type depository.** KUM (holotype); NIAES (paratype).

**Type locality.** Philippines (Luzon Is.: Ilocos Norte Prov., Pagudpud).

**Distribution.** Philippines: Luzon Is. (Cagayan, Ilocos Norte, Nueva Vizcaya) (Yoshitake 2019c; Rukmane-Bārbale and Cabras 2021).

***Pachyrhynchusforsteni* Vollenhoven, 1864**

**Type depository.** Unknown.

**Type locality.** Indonesia (Molucas Is.: North Maluku Prov., Ternate).

**Distribution.** Indonesia: Maluku Isls. (Halmahera Is., Makian Is., Molucas Is., Ternate Is., Morotai Is.) (Yoshitake 2018b; Rukmane 2019b; Bollino 2023).

**Remarks.** According to the original description of *P.forsteni* by Snellen van Vollenhoven (1864), the distribution of this species included Ternate, Halmahera, and Sumatra. However, the record from Sumatra is now questioned due to geographical discrepancies (Yoshitake 2018b).

***Pachyrhynchusfranciscoi* Rukmane & Cabras, 2018**

**Type depository.**DUBC (holotype, paratype).

**Type locality.** Philippines (Panay Is.: Antique Prov., Culasi, Aklan).

**Distribution.** Philippines: Panay Is. (Antique, Culasi, Aklan).

**Remarks.** This species is very similar and sympatric with *P.felipeae*Rukmane and Cabras (2018b) from Panay Island, Philippines.

***Pachyrhynchusgaleraensis* Schultze, 1934**

**Type depository.**SMTD (holotype).

**Type locality.** Philippines (Mindoro Is.: Puetro Galera).

**Distribution.** Philippines: Mindoro Is. (Puetro Galera).

***Pachyrhynchusgemmatusgemmatus* Waterhouse, 1841**

Pachyrhynchusgemmatusvar.atratus Heller, 1912: 308. Synonymized by Schultze (1924: 329).

**Type depository.**NHMUK (syntype), SMTD (*).

**Type locality.** Philippines.

**Distribution.** Philippines: Luzon Is. (Aurora; Calayan, Sanchez Mira, Santa Ana.; Moutain Prov.; Manila; Nueva Vizcaya; Isabela; Quirino) (Waterhouse 1841; Schultze 1924; Rukmane 2018c, 2019b; Rukmane-Bārbale and Cabras 2021).

***Pachyrhynchusgemmatuspurpureus* Kraatz, 1888**

**Type depository.**SMTD (*).

**Type locality.** Philippines (Luzon Is.).

**Distribution.** Philippines: Luzon Is. (Cagayan, Sanchez Mira) (Schultze 1924).

***Pachyrhynchusgilvomaculatus* Yoshitake, 2017**

**Type depository.**NIAES (holotype); KUM, MBLI (paratypes).

**Type locality.** Philippines (Mindanao Is.: South Cotabato Prov., Mt. Matutum).

**Distribution.** Philippines: Mindanao Is. (Soccsksargen region, South Cotabato Prov.) (Yoshitake 2017c).

***Pachyrhynchusgloriosusgloriosus* Faust, 1895**

**Type depository.**SMTD (*).

**Type locality.** Philippines (Luzon Is.).

**Distribution.** Philippines: Luzon Is. (Aurora; Ifugao; Isabela; Kalinga; Laguna, Mt. Banahao; Nueva Vizcaya; Rizal) (Faust 1895a; Schultze 1924; Rukmane-Bārbale and Cabras 2021).

**Remarks.** A specimen examined by Rukmane (2019b) was recorded from Negros Island, a locality that differs from previously known distribution records, which have been confined to Luzon. While this may be a special record, its validity as a confirmed observation requires further verification.

***Pachyrhynchusgloriosusabbreviatus* Schultze, 1922**

**Type depository.**SMTD (*).

**Type locality.** Philippines (Luzon Is.: Mountain Prov., Bontoc).

**Distribution.** Philippines: Luzon Is. (Mountain Prov., Bontoc; Kalinga, Lubuagan) (Schultze 1922b; Schultze 1924).

***Pachyrhynchushalconensis* Schultze, 1922**

**Type depository.**SMTD (syntypes).

**Type locality.** Philippines (Mindoro Is.: Mt. Halcon).

**Distribution.** Philippines: Mindoro Is. (Mt. Halcon) (Schultze 1922b; Schultze 1923; Rukmane 2019b; Rukmane-Bārbale and Cabras 2021).

***Pachyrhynchushelenperrinae* Rukmane, 2018**

**Type depository.**MNHN (holotype, paratypes).

**Type locality.** Philippines (Ch. Semper).

**Distribution.** Philippines: Mindanao Is. or Mindanao PAIC.

**Remarks.**Rukmane (2018d) mentioned that species on a particular island share common features, which are clearly distinct from those of species found on another island. Therefore, based on their general appearance, this species might be distributed across Mindanao Island or the Mindanao PAIC (Rukmane 2018d).

***Pachyrhynchushelleri* Kuntzen, 1914**

**Type depository.**ZMHB (holotype).

**Type locality.** Philippines (Luzon Is.).

**Distribution.** Philippines: Luzon Is. (Schultze 1924).

***Pachyrhynchushirokii* Yoshitake, 2012**

**Type depository.**NIAES (holotype, paratypes); PCKS (paratypes).

**Type locality.** Philippines (Mindanao Is.: Mt. Apo).

**Distribution.** Philippines: Mindanao Is. (Bukidnon; Cotabato; Davao del Sur; Lanao del Sur) (Rukmane 2019b; Rukmane-Bārbale and Cabras 2021).

***Pachyrhynchusigorota* Schultze, 1917**

**Type depository.**SMTD (syntypes)

**Type locality.** Philippines (Luzon Is.: Benguet Prov., Haight’s Place).

**Distribution.** Philippines: Luzon Is. (Benguet, Mt. Pawai, Mt. Pulog; Ifugao; Isabela, Mt. Moises; Mountain Prov., Mt. Polis; Nueva Vizcaya) (Schultze 1924; Rukmane 2019c; Rukmane-Bārbale and Cabras 2021).

***Pachyrhynchusilgas* Rukmane, 2017**

**Type depository.**DUBC (holotype).

**Type locality.** Philippines (Samar Is.: Northern Samar Prov., Lope De Vega).

**Distribution.** Philippines: Samar Is. (Northern Samar) (Rukmane 2017).

***Pachyrhynchusimitans* Rukmane & Bollino, 2020**

**Type depository.**SMTD (holotype); DUBC, MZZC, MBLI (paratypes).

**Type locality.** Philippines (Mindanao Is.: Zamboanga City, Labuan).

**Distribution.** Philippines: Mindanao Is. (Zamboanga City, Zamboanga del Norte, Zamboanga Sibugay).

***Pachyrhynchusinclytus* Pascoe, 1873**

*Pachyrhynchusignipes* Chevrolat, 1881b: 359. Synonymized by Schultze (1924: 339).

*Pachyrhynchusmodestior* Behrens, 1887: 240. Synonymized by Schultze (1924: 339).

P.modestiorvar.apicalis Kraatz, 1888: 26. Synonymized by Schultze (1924: 340).

P.modestiorvar.transversatus Heller, 1921: 544. Synonymized by Schultze (1924: 340).

**Type depository.**NHMUK (syntype), SMTD (*).

**Type locality.** Philippines.

**Distribution.** Philippines: Luzon Is. (Benguet, Mt. Trail, Baguio, Mt. Mirador, Mt. Santo Tomas, Trinidad; Ifugao; Manila; Mountain Prov.; Nueva Vizcaya, Imugan) (Schultze 1924; Rukmane 2019b, 2019c; Rukmane-Bārbale and Cabras 2021).

***Pachyrhynchusinfernalis* Fairmaire, 1897**

*Pachyrhynchusniger* Sakaguchi, 1927: 24. Synonymized by Rukmane (2018a: 65).

**Type depository.** Unknown.

**Type locality.** Japan (Ryukyu, Ishigaki Is.).

**Distribution.** Japan: Ryukyu Isls. (Iriomote Is., Ishigaki Is.) (Schultze 1923; Starr and Wang 1992).

**Host plants.***Mangiferaindica* (Anacardiaceae) and *Glochidion* sp. (Euphorbiaceae) (Oshiro 1991).

**Remarks.***Pachyrhynchusinfernalis* is known from Ryukyu Islands. However, the identification of a specimen from “Bangsamoro” as *P.infernalis* by Rukmane-Bārbale and Cabras (2021) from the DUBC collection might need to be reevaluated.

***Pachyrhynchusjitanasaius* Chen & Lin, 2017**

**Type depository.** NMNS (holotype, paratypes).

**Type locality.** Taiwan (Taitung County, Ludao Is.).

**Distribution.** Taiwan: Ludao Is.

**Host plants.** Among all the individuals found on the host plants, 50% were found on Shoebutton ardisia (*Ardisiaelliptica*, Primulaceae), 48% on beef wood (*Casuarinaequisetifolia*, Casuarinaceae), and 2% on litsea (*Litseaacutivens*, Lauraceae) (Chen et al. 2017).

***Pachyrhynchusjugifer* Waterhouse, 1841**

*Pachyrhynchusrhodopterus* Chevrolat, 1841: 224. Synonymized by Schultze (1923: 653).

**Type depository.**NHMUK (holotype).

**Type locality.** Philippines.

**Distribution.** Philippines: Panay Is. (Capiz, Aklan, Jamindan, Mt. Macosolon) (Waterhouse 1841; Schultze 1923; Rukmane 2019b; Rukmane-Bārbale and Cabras 2021).

**Remarks.** Four specimens examined by Rukmane (2018c) were recorded from Luzon Island (Manila). However, other specimens were found on Panay Island.

***Pachyrhynchuskirklayroni* Rukmane, 2019**

**Type depository.**DUBC (holotype).

**Type locality.** Philippines (Luzon Is.: Ilocos Norte Prov., Adams).

**Distribution.** Philippines: Luzon Is. (Ilocos Norte) (Rukmane 2019d).

***Pachyrhynchuskraslavae* Rukmane & Barševskis, 2016**

**Type depository.**DUBC (holotype).

**Type locality.** Philippines (Mindanao Is.: Davao de Oro Prov., Mabini).

**Distribution.** Philippines: Mindanao Is. (Agusan del Sur, San Francisco; Surigao del Norte) (Rukmane 2019a).

***Pachyrhynchuslacunosus* Heller, 1912**

**Type depository.**NHMUK (syntype); SDEI (syntype).

**Type locality.** Philippines.

**Distribution.** Philippines: Luzon Is. (Benguet, Mt. Pulogloko; Ifugao, Asipulo, Banaue, Hungduan, Tinok; Isabela; Nueva Viscaya, Ambaguio, Belance, Dupax Del Sur, Kasibu, Kayapa, Malico, Sta. Fe.; Quezon) (Schultze 1924; Rukmane-Bārbale 2020b; Rukmane-Bārbale and Cabras 2021).

***Pachyrhynchuslatifasciatus* Waterhouse, 1842**

**Type depository.**NHMUK (holotype).

**Type locality.** Philippines.

**Distribution.** Philippines: Samar Is. (Northern Samar, Lope de Vega) (Waterhouse 1842; Yoshitake 2017c; Rukmane-Bārbale and Cabras 2021).

***Pachyrhynchuslayroni* Rukmane & Cabras, 2018**

**Type depository.**DUBC (holotype, paratypes).

**Type locality.** Philippines (Panay Is.: Antique Prov., Madja-as).

**Distribution.** Philippines: Panay Is. (Antique) (Rukmane and Cabras 2018b).

***Pachyrhynchuslibucanus* Schultze, 1923**

**Type depository.**SMTD (holotype).

**Type locality.** Philippines (Libucan Is.: near Samar).

**Distribution.** Philippines: Libucan Is.

***Pachyrhynchusloheri* Schultze, 1917**

**Type depository.**SMTD (holotype).

**Type locality.** Philippines (Luzon Is.: Bulacan Prov., Mount Guinuisan).

**Distribution.** Philippines: Luzon Is. (Bulacan, Mount Guinisan) (Schultze 1924; Rukmane-Bārbale 2020b).

***Pachyrhynchuslorquini* Chevrolat, 1881**

*Pachyrhynchusflavopunctatus* Kraatz, 1888: 30. Synonymized by Schultze (1924: 334).

*Pachyrhynchusflavomaculatus* Kraatz, 1888: 32. Synonymized by Schultze (1924: 334).

**Type depository.**NHRS (syntypes).

**Type locality.** Philippines.

**Distribution.** Philippines: Luzon Is. (Laguna, Mt. Maquiling); Marinduque Is. (Mt. Malindig) (Schultze 1924; Rukmane 2018c; Rukmane 2019b).

**Remarks.** In the original description of *P.lorquini*, the type specimen was recorded from Maldonado (Chevrolat 1881b), but subsequent findings were in Luzon. The actual distribution range of the species needs to be re-evaluated.

***Pachyrhynchuslubanganus* Bollino & Sandel, 2015**

**Type depository.**SMTD (holotype); MBLI, CFS (paratypes).

**Type locality.** Philippines (Lubang Is.: south-west of Tilik).

**Distribution.** Philippines: Lubang Is.

***Pachyrhynchusmarinduquensis* Rukmane & Barševskis, 2016**

**Type depository.**DUBC (holotype, paratype).

**Type locality.** Philippines (Marinduque Is.: Marinduque Prov., Buenavista).

**Distribution.** Philippines: Marinduque Is. (Marinduque, Buenavista, Boac; Mt. Malindig) (Rukmane-Bārbale and Cabras 2021).

***Pachyrhynchusmaruyamai* Yoshitake, 2019**

**Type depository.**NIAES (holotype); MCKUM (paratypes).

**Type locality.** Philippines (Catanduanes Is.: San Miguel, Kilikilihan).

**Distribution.** Philippines: Catanduanes Is. (Yoshitake 2019f).

***Pachyrhynchusmasatoshii* Yoshitake & Yap, 2017**

**Type depository.**UPLB (holotype).

**Type locality.** Philippines (Luzon Is.: Quezon Prov., Dolores, Brgy. Kinabuhayan, Mt. Banahaw).

**Distribution.** Philippines: Luzon Is. (Calabarzon regio) (Yoshitake and Yap 2017).

***Pachyrhynchusmasatoshiensis* Rukmane-Bārbale, 2024**

**Type depository.**DUBC (holotype, paratypes).

**Type locality.** Philippines (Luzon Is.: Albay Prov., Tabaco, Mayon Volcano).

**Distribution.** Philippines: Luzon Is. (Quezon, Gen Nakar) (Rukmane-Bārbale 2024).

***Pachyrhynchusmiltoni* Cabras & Rukmane, 2016**

**Type depository.**CMU-MZ (holotype, paratype).

**Type locality.** Philippines (Mindanao Is.: Davao del Sur Prov., Davao City, Marilog District).

**Distribution.** Philippines: Mindanao Is. (Davao del Sur) (Mohagan et al. 2018; Rukmane-Bārbale and Cabras 2021; Cabras et al. 2022a).

**Host plants.** This species has been documented on *Melastomamalabathricum* (Melastomataceae), *Piperaduncum* (Piperaceae), *Lithocarpusboholensis* (Fagaceae), *Theobromacacao* (Malvaceae), *Atunaracemosa* (Chrysobalanaceae), and *Helianthus* sp. (Asteraceae) (Cabras et al. 2022a).

***Pachyrhynchusmindoroensis* Rukmane & Háva, 2020**

**Type depository.**DUBC (holotype, paratypes); JHAC (paratype).

**Type locality.** Philippines (Mindoro Is.: Oriental Mindoro Prov., Puerto Galera).

**Distribution.** Philippines: Mindoro Is. (Oriental Mindoro, Baco, Puerto Galera, Bacon, Mt. Halcon).

***Pachyrhynchusmohagani* Bollino & Sandel, 2015**

**Type depository.**SMTD (holotype); MBLI, CFS (paratypes).

**Type locality.** Philippines (Lubang Is.: south-west of Tilik).

**Distribution.** Philippines: Lubang Is.

***Pachyrhynchusmollendorffimollendorffi* Heller, 1898**

**Type depository.**SMTD (holotype).

**Type locality.** Philippines.

**Distribution.** Philippines: Panay Is. (Antique) (Rukmane and Cabras 2018a).

***Pachyrhynchusmollendorffimarinduquanus* Rukmane & Cabras, 2018**

**Type depository.**DUBC (holotype, paratypes).

**Type locality.** Philippines (Marinduque Is.: Marinduque Prov., Buenavista).

**Distribution.** Philippines: Marinduque Is. (Marinduque, Buenavista, Mt. Malinding) (Rukmane and Cabras 2018a).

***Pachyrhynchusmoniliferusmoniliferus* Germar, 1824**

*Pachyrhynchusconfinis* Chevrolat, 1841: 226. Synonymized by Schultze (1923: 634).

**Type depository.**SMTD (*).

**Type locality.** Philippines (Luzon Is.: Manila).

**Distribution.** Philippines: Luzon Is. (Aurora, Dingalan; Cagayan; Ifugao, Banaue; Quirino; Nueva Vizcaya, Belance; Isabela; Mountain Prov.; Kalinga, Pinukpuk; Benguet; Camarines; Batangas; Laguna, Los Baños, Mt. Makiling, Mt. Banahao; Rizal, Bosoboso, Montalban, Taytay, Antipolo; Manila; Paete, Bataan, Limay); Mindoro Is. (Mt. Halcon); Marinduque Is.; Bohol Is.; Negros Is.; Camiguin Is.; Calayan Is.; Catanduanes Is. (Schultze 1923; Rukmane 2018c, 2018e, 2019b, 2019c; Rukmane-Bārbale and Cabras 2021).

**Host plant.** Cocoa, *Theobromacacao* (Kayashima 1940); Bishop Wood, *Bischofiajavanica* (Phyllanthaceae) (this study).

**Remarks.** The larvae of this species were found to feed on cacao fruit imported from the Philippines to Taiwan (Kayashima 1940). In addition, it is considered as one of the insect pests of cocoa plantations in the Philippines, especially in Luzon (Domingo et al. 2023).

***Pachyrhynchusmoniliferusabranus* Heller, 1934**

**Type depository.**SMTD (*).

**Type locality.** Philippines (Luzon Is.: Abra Prov.).

**Distribution.** Philippines: Luzon Is. (Abra, Nueva Vizcaya); Mindoro Is. (Baco; Mt. Halcon) (Rukmane 2018e).

**Remarks.**Heller (1934) treated *P.stellulifer* as a distinct species, and described *abranus* as its subspecies simultaneously. However, Heller described *stellulifer* as a variety (subspecies) of *P.moniliferus* in 1912. This classification has been followed by Schultze (1924, 1934) and Rukmane (2018a, 2018e). There has been no further related study since then until Rukmane (2018a, e) listed *abranus* as a subspecies of *P.moniliferus*, which is followed herein. Rukmane (2018e) incorrectly spelled “abranus” as “eburnus”, and the specimens from Mindoro Island might need to be evaluated in the future.

***Pachyrhynchusmoniliferusbabuyanensis* Rukmane, 2018**

**Type depository.**DUBC (holotype, paratypes).

**Type locality.** Philippines (Babuyan Is.).

**Distribution.** Only known from the type locality.

***Pachyrhynchusmoniliferuschevrolati* Eydoux & Souleyet, 1839**

*Pachyrhynchuschlorolineatus* Waterhouse, 1841: 25. Synonymized by Schultze (1923: 644).

*Pachyrhynchusmandarinus* Chevrolat, 1841: 226. Synonymized by Schultze (1923: 644).

*Pachyrhynchusconcinnus* Waterhouse, 1842: 70. Synonymized by Schultze (1923: 644).

**Type depository.**SMTD (*).

**Type locality.** Philippines.

**Distribution.** Philippines: Catanduanes Is. (Catanduanes Prov., Virac); Luzon Is. (Aurora, Dingalan; Isabela; Quirino; Manila; Tayabas; Albay; Polillo); Samar Is. (Catarman, Borongan); Mindanao Is. (Misamis Occidental, Labuyo) (Schultze 1923; Rukmane 2018c, 2018d, 2018e).

***Pachyrhynchusmoniliferusherbidus* Rukmane, 2018**

**Type depository.**DUBC (holotype); SMTD (paratypes).

**Type locality.** Philippines (Samar Is.: Northern Samar, Lope De Vega).

**Distribution.** Only known from the type locality.

***Pachyrhynchusmoniliferusjagori* Heller, 1912**

**Type depository.**SMTD (*).

**Type locality.** Philippines (Samar Is.).

**Distribution.** Philippines: Samar Is.; Catanduanes Is. (Rukmane 2018e).

**Remarks.** The type locality of this subspecies is Samar Island, Philippines (Heller 1912). However, Rukmane (2018e) examined a specimen from Catanduanes Island, which requires further clarification.

***Pachyrhynchusmoniliferusstellulifer* Heller, 1912**

**Type depository.**SMTD (*).

**Type locality.** Philippines.

**Distribution.** Philippines: Luzon Is. (Benguet, Ilocos Sur, Pangasinan); ​​Mindoro Is. (Naujan; Mangaran; San Jose; Mt. Halcon; Puerto Galera) (Schultze 1923, 1934).

***Pachyrhynchusmorio* Heller, 1912**

**Type depository.**NHMUK (syntype); SMTD (syntypes).

**Type locality.** Philippines (Luzon Is.).

**Distribution.** Philippines: Luzon Is. (Benguet, Mt. Lusong) (Schultze 1924).

***Pachyrhynchusmorotaiensis* Vollenhoven, 1864**

*Pachyrhynchuswaterhousei* Faust, 1895b: 95. Synonymized by Schultze (1923: 661).

**Type depository.**SMTD (syntypes).

**Type locality** Indonesia (Morotai).

**Distribution.** Indonesia: Maluku Isls. (Morotai, Moluccas) (Faust 1895b; Schultze 1923; Rukmane 2018c).

***Pachyrhynchusmultipunctatusmultipunctatus* Waterhouse, 1841**

*Pachyrhynchusauroguttatus* Chevrolat, 1881: 348. Synonymized by Schultze (1924: 316).

**Type depository.** Unknown.

**Type locality.** Philippines.

**Distribution.** Philippines: Bohol Is. (Bohol, Sevilla); Luzon Is. (Nueva Vizcaya; Manila) (Waterhouse 1841; Schultze 1924; Yoshitake 2018c; Rukmane 2019b; Rukmane-Bārbale and Cabras 2021).

***Pachyrhynchusmultipunctatusendoi* Yoshitake, 2018**

**Type depository.**NIAES (holotype, paratypes); MCKUM (paratypes).

**Type locality.** Philippines (Cebu Is.: Cebu city, Barangay Busay, Sitio Rosvelt).

**Distribution.** Only known from the type locality (Yoshitake 2018c).

***Pachyrhynchusnaokii* Yoshitake, 2012**

**Type depository.**NIAES (holotype).

**Type locality.** Philippines (Mindanao Is.: Mt. Syniop).

**Distribution.** Philippines: Mindanao Is. (Cotabato, Sultan Kudarat; Lanao del Sur) (Rukmane-Bārbale and Cabras 2021).

***Pachyrhynchusnaokoae* Yoshitake, 2019**

*Pachyrhynchussibuyanensis*Rukmane 2019b: 42. Synonymized by Yoshitake (2019a: 408).

**Type depository.**NIAES (holotype, paratypes); KUM (paratypes).

**Type locality.** Philippines (Sibuyan Is.: Mimaropa region, Romblon Prov.).

**Distribution.** Only known from the type locality.

***Pachyrhynchusnegrosensis* Schultze, 1924**

**Type depository.**SMTD (holotype).

**Type locality.** Philippines (Negros Is.: Occidental Negros Prov., Cuernos Mountains).

**Distribution.** Philippines: Negros Is. (Occidental Negros Prov., Mt. Canlaon, Benedicto) (Rukmane 2019b; Rukmane-Bārbale and Cabras 2021).

***Pachyrhynchusneoabsurdus* Rukmane, 2017**

**Type depository.**DUBC (holotype, paratype).

**Type locality.** Philippines (Mindanao Is.: Bukidnon Prov., Mt. Kalatungan).

**Distribution.** Philippines: Mindanao Is. (Bukidnon, Intavas) (Rukmane 2017).

***Pachyrhynchusniisatoi* Yoshitake, 2017**

**Type depository.** KUM (holotype); KUM, MBLI, NIAES (paratypes).

**Type locality.** Philippines (Luzon Is.: Quirino Prov., Nagutipunan).

**Distribution.** Philippines: Luzon Is. (Central Luzon; Cagayan Valley region; Aurora; Nueva Vizcaya, Kayapa, Kasibu, Dupax Del Sur; Quirino, Madela, Nagtipunan; Isabela; Aurora; Rizal) (Yoshitake 2017d; Rukmane-Bārbale 2020b; Rukmane-Bārbale and Cabras 2021).

***Pachyrhynchusnitcisi* Rukmane & Barševskis, 2016**

**Type depository.**DUBC (holotype, paratypes).

**Type locality.** Philippines (Mindanao Is.: Sarangani Prov., Malungon).

**Distribution.** Philippines: Mindanao Is. (Agusan; Agusan del Sur, Sibagat; Bukidnon; Cotabato; Sarangani, Malungon) (Rukmane-Bārbale and Cabras 2021).

***Pachyrhynchusnobilisnobilis* Heller, 1912**

**Type depository.**NHMUK (syntype); SMTD (syntype); SDEI (syntype).

**Type locality.** Philippines.

**Distribution.** Philippines: Babuyan Islands (Babuyan Claro; Calayan Is.); Luzon Is. (Ifugao; Isabela; Moutain Prov.; Nueva Vizcaya; Quirino) (Rukmane 2019b; Rukmane-Bārbale and Cabras 2021; Tseng et al. 2021).

***Pachyrhynchusnobilisyamianus* Kano, 1929**

**Type depository.** Unknown.

**Type locality.** Taiwan (Taitung County, Lanyu Is. (= Kotosho)).

**Distribution.** Taiwan: Lanyu Is.; Ludao Is.

**Host plants.***Epipremnumpinnatum* (Araceae) (this study).

**Remarks.***Pachyrhynchusnobilisyamianus* was initially considered a distinct species as *P.yamianus* Kano, 1929. However, Schultze (1937) examined the specimens and regard it as a subspecies of *P.nobilis*. Whether *P.nobilisyamianus* is a distinct species requires re-examination.

***Pachyrhynchusnoeli* Yoshitake, 2019**

**Type depository.** KUM (holotype).

**Type locality.** Philippines (Sibuyan Is.: Mimaropa region, Romblon Prov.).

**Distribution.** Only known from the type locality (Yoshitake 2019e).

***Pachyrhynchusnotocruciatus* Yoshitake, 2017**

**Type depository.**NIAES (holotype).

**Type locality.** Philippines (Mindanao Is.: Mt. Apo).

**Distribution.** Philippines: Mindanao Is. (Cotabato, Alamada; Mt. Apo) (Yoshitake 2017c; Rukmane-Bārbale and Cabras 2021).

***Pachyrhynchusobumanuvu* Cabras, Donato, Medina, & Van Dam, 2021**

**Type depository.**PNMNH (holotype); UMCRC, CASENT, MBLI (paratypes).

**Type locality.** Philippines (Mindanao Is.: Davao City).

**Distribution.** Philippines: Mindanao Is. (Bukidnon, Davao city) (Cabras et al. 2021, 2022a).

**Host plants.***Procrisurdanetensis* (Urticaceae) and *Elatostema* sp. (Urticaceae). (Cabras et al. 2021, 2022a)

***Pachyrhynchusoccidentalis* Rukmane, 2017**

**Type depository.**DUBC (holotype, paratypes).

**Type locality.** Philippines (Mindanao Is.: Davao Del Sur Prov., Kapatagan).

**Distribution.** Philippines: Mindanao Is. (Cotabato, Mt. Parke; Davao Del Sur, Mt. Apo; Sarangani, Kiamba) (Rukmane 2017; Háva and Rukmane 2018; Rukmane 2019b; Yoshitake and Bollino 2019; Rukmane-Bārbale and Cabras 2021).

***Pachyrhynchusochroplagiatusochroplagiatus* Heller, 1912**

**Type depository.**SMTD (holotype).

**Type locality.** Philippines (Luzon Is.: Benguet Prov., Mt. Pulog).

**Distribution.** Philippines: Luzon Is. (Aurora; Benguet, Mt. Pulog; Nueva Vizcaya) (Schultze 1924; Rukmane-Bārbale and Cabras 2021).

***Pachyrhynchusochroplagiatusmultiplagiatus* Schultze, 1924**

**Type depository.**SMTD (*).

**Type locality.** Philippines.

**Distribution.** Philippines: Luzon Is. (Rukmane 2018a).

***Pachyrhynchusoctoannulatus* Yoshitake, Bollino & Sandel, 2019**

**Type depository.**NIAES (holotype, paratypes); KUM, CFS, MBLI (paratypes).

**Type locality.** Philippines (Mindanao Is.: Lanao del Sur Prov., Wao).

**Distribution.** Philippines: Mindanao Is. (Bukidnon; Bangsamoro Autonomous Region ni Muslim Mindanao; Soccksargen region; Lanao del Sur, Wao; Cotabato) (Rukmane 2019a; Yoshitake et al. 2019b).

***Pachyrhynchusohbayashii* Yoshitake, 2017**

**Type depository.**NIAES (holotype).

**Type locality.** Indonesia (Biak Is.: Irian Jaya, North of Biak).

**Distribution.** Only known from the type locality (Yoshitake 2017a).

***Pachyrhynchusorbiferorbifer* Waterhouse, 1841**

*Pachyrhynchusalboguttatus* Chevrolat, 1841: 226. Synonymized by Schultze (1923: 638).

*Pachyrhynchusfahrei* Schoenherr, 1845: 388. Synonymized by Schultze (1923: 638).

*Pachyrhynchusfimbriatus* Chevrolat, 1841: 224. Synonymized by Schultze (1923: 637).

*Pachyrhynchusglobulipennis* Chevrolat, 1841: 225. Synonymized by Schultze (1923: 637).

*Pachyrhynchuspretiosus* Chevrolat, 1841: 225. Synonymized by Schultze (1923: 637).

*Pachyrhynchusscintillans* Chevrolat, 1841: 225. Synonymized by Schultze (1923: 637).

**Type depository.**NHRS (syntypes); SMTD (*).

**Type locality.** Philippines.

**Distribution.** Philippines: Luzon Is. (Aurora; Benguet, Baguio to Bontoc, Bued River Valley, Baguio, Mt. Santo Tomas, Mount Mirador, Trinidad, Mountain Trail; Ifugao; Ilocos Norte, Bangui, Burgos, Mount Nagapatan; Isabela; Mountain Prov.; Nueva Viscaya; Quirino; Kalinga, Lubuagan; Cagayan, Sanchez Mira; Manila) (Waterhouse 1841; Schultze 1923; Rukmane and Barševskis 2017; Rukmane 2018c, 2019b, 2019c).

**Remarks.** One female was found in Ako (Pingtung, Taiwan) (Kôno 1930); however, no record was found after that.

***Pachyrhynchusorbiferardens* Chevrolat, 1841**

**Type depository.**SMTD (*).

**Type locality.** Philippines.

**Distribution.** Philippines: Luzon Is. (Cagayan, Sanchez Mira) (Schultze 1923; Rukmane 2018a).

***Pachyrhynchusorbiferazureus* Schultze, 1922**

**Type depository.**SMTD (*).

**Type locality.** Philippines (Luzon Is.: Benguet Prov., Kabayan).

**Distribution.** Philippines: Luzon Is. (Benguet, Kabayan; Cagayan; Isabela; Kalinga) (Schultze 1922b; Schultze 1923; Rukmane and Barševskis 2017).

***Pachyrhynchusorbifercallainus* Yoshitake, 2017**

**Type depository.**NIAES (holotype, paratypes).

**Type locality.** Philippines (Luzon Is.: Cagayan Prov., Sierra Madre).

**Distribution.** Philippines: Luzon Is. (Cagayan Valley region) (Yoshitake 2017b).

***Pachyrhynchusorbifercirculifer* Chevrolat, 1841**

**Type depository.**SMTD (*).

**Type locality.** Philippines.

**Distribution.** Philippines: Luzon Is. (Apayao; Cagayan; Mountain Prov.; Ilocos Norte, Bangui; Isabela; Quirino; Nueva Vizcaya) (Schultze 1923; Rukmane and Barševskis 2017).

***Pachyrhynchusorbifergemmans* Chevrolat, 1841**

**Type depository.**NHRS (syntypes); SMTD (*).

**Type locality.** Philippines (Luzon Is.: Manila).

**Distribution.** Philippines: Luzon Is. (Apoyao; Cagayan, Tuao, Rio Chico; Isabela, Mt. Moises; Ifugao; Mountain Prov.; Quirino; Laguna; Manila) (Rukmane and Barševskis 2017; Rukmane 2018c, 2019c).

***Pachyrhynchusorbiferinornatus* Waterhouse, 1841**

**Type depository.**SMTD (*).

**Type locality.** Philippines.

**Distribution.** Philippines: Calayan Is.; Luzon Is. (Cagayan, Ilocos Norte) (Waterhouse 1841; Schultze 1923).

***Pachyrhynchusorbifermurinus* Heller, 1934**

**Type depository.**SMTD (*).

**Type locality.** Philippines (Luzon Is.: Ilocos Sur Prov., Cabugao).

**Distribution.** Philippines: Luzon Is. (Apayao; Ilocos Sur; Mountain Prov.; Quirino) (Rukmane and Barševskis 2017).

***Pachyrhynchusorbiferstriatomaculatus* Yoshitake, 2017**

**Type depository.**NIAES (holotype, paratypes); KUM (paratypes).

**Type locality.** Philippines (Luzon Is.: Nueva Vizcaya Prov., Aritao).

**Distribution.** Philippines: Luzon Is. (Cagayan Valley region) (Yoshitake 2017c).

***Pachyrhynchusorientalis* Rukmane, 2017**

**Type depository.**DUBC (holotype, paratypes).

**Type locality.** Philippines (Mindanao Is.: Bukidnon Prov., Cabanglasan).

**Distribution.** Philippines: Mindanao Is. (Bukidnon, Cabanglasan; Surigao Del Sur, San Miguel) (Rukmane 2017).

***Pachyrhynchusottomerkli* Rukmane, 2019**

**Type depository.** HUF (holotype, paratypes).

**Type locality.** Philippines (Mindanao Is.: Bukidnon Prov., Maluko).

**Distribution.** Only known from the type locality (Rukmane 2019c).

***Pachyrhynchuspanumanon* Cabras & Medina, 2022**

**Type depository.**PNMNH (holotype); UMCRC (paratypes).

**Type locality.** Philippines (Mindanao Is.: Misamis Oriental Prov., Gingoog City).

**Distribution.** Only known from the type locality.

**Host plants.***Glochidion* sp. (Phyllanthaceae) and *Clethra* sp. (Clethraceae) (Cabras et al. 2022b).

***Pachyrhynchusperpulcher* Waterhouse, 1841**

**Type depository.**NHMUK (holotype).

**Type locality.** Philippines.

**Distribution.** Philippines: Babuyan Is.; Batanes Is.; Calayan Is.; Camiguin Is.; Luzon Is. (Cagayan; Ilocos Norte; Nueva Vizcaya; Kalinga, Pinukpuk; Quirino) (Waterhouse 1841; Schultze 1924; Rukmane 2019b; Rukmane-Bārbale and Cabras 2021; Tseng et al. 2021).

***Pachyrhynchusphaleratusphaleratus* Waterhouse, 1841**

*Pachyrhynchuselenae* Rukmane, 2016: 82. Synonymized by Yoshitake, Bollino and Sandel (2019a: 197).

**Type depository.**NHMUK (holotype).

**Type locality.** Philippines (Catanduanes Is.: Virac).

**Distribution.** Philippines: Catanduanes Is. (Virac); Luzon Is. (Aurora; Isabela; Quirino) (Waterhouse 1841; Schultze 1923; Yoshitake et al. 2019a).

***Pachyrhynchusphaleratusbadiovittatus* Yoshitake, 2019**

**Type depository.** KUM (holotype).

**Type locality.** Philippines (Marinduque Is.: Marinduque Prov., Gasan, Tiguion).

**Distribution.** Only known from the type locality.

***Pachyrhynchusphaleratusdannylayroni* Rukmane, 2019**

**Type depository.**DUBC (holotype).

**Type locality.** Philippines (Luzon Is.: Aurora Prov., Dingalan).

**Distribution.** Only known from the type locality (Rukmane 2019d).

***Pachyrhynchuspinorumpinorum* Pascoe, 1873**

Pachyrhynchuspinorumvar.dimidiatus Heller, 1912: 306. Synonymized by Schultze (1924: 351).

**Type depository.**NHMUK (syntype), SMTD (*).

**Type locality.** Philippines (Luzon Is.).

**Distribution.** Philippines: Luzon Is. (Mt. Makiling; Benguet, Mt. Trail, Baguio, Atoc, Tublay, La Trinidad; Ifugao, Banaue, Hungduan; Moutain Prov., Barlig; Nueva Vizcaya, Kayapa, Sta. Fe., Dupax) (Schultze, 1920; Rukmane 2019b, 2019c; Rukmane-Bārbale 2020b; Rukmane-Bārbale and Cabras 2021).

***Pachyrhynchuspinorumtransversalis* Heller, 1912**

**Type depository.**SMTD (*).

**Type locality.** Philippines.

**Distribution.** Philippines: Luzon Is.

***Pachyrhynchuspostpubescenspostpubescens* Schultze, 1922**

**Type depository.**SMTD (syntypes).

**Type locality.** Philippines (Mindanao Is.: Bukidnon Prov., Lindabon).

**Distribution.** Philippines: Bohol Is.; Luzon Is. (Bukidnon, Mt. Kiamo, Cabanglasan, Intavas, Kalatungan, Panamokan, San Fernando); Mindanao Is. (Davao del Sur, Mt. Apo) (Schultze 1922b; Cabras et al. 2017; Rukmane 2019a, 2019b; Rukmane-Bārbale and Cabras 2021).

***Pachyrhynchuspostpubescensconfluens* Janczyk, 1959**

**Type depository.** Unknown.

**Type locality.** Philippines (Mindanao Is.).

**Distribution.** Only known from the type locality.

***Pachyrhynchuspseudamabilis* Yoshitake, 2012**

**Type depository.**NIAES (holotype, paratypes).

**Type locality.** Philippines (Mindanao Is.: Mt. Apo).

**Distribution.** Philippines: Mindanao Is. (Mt. Apo; Agusan del Sur, Sibagat; Bukidnon, Cabanglasan, Panamokan, Mt. Kalatungan; Cotabato, Mt. Parke, Kidapawan; Davao del Sur, Davao City, Marilog District, Catigan, Toril, Calinan, Kapatagan; Lanao del Sur, Wao; Misamis Oriental, Mat-i, Claveria; Sarangani, Kiamba) (Cabras 2020; Rukmane-Bārbale and Cabras 2021; Cabras et al. 2022a).

**Host plants.***Theobromacacao* (Malvaceae), *Crotonleiophyllus* (Euphorbiaceae), *Piperaduncum* (Piperaceae), and *Lithocarpusboholensis* (Fagaceae) (Cabras et al. 2022a).

***Pachyrhynchuspseudhalconensis* Rukmane, 2016**

**Type depository.**DUBC (holotype, paratypes).

**Type locality.** Philippines (Mindoro Is.: Mindoro Oriental Prov., Puerto Galera).

**Distribution.** Philippines: Mindoro Is. (Mindoro Oriental Prov., Puerto Galera, Mt. Halcon, Baco).

***Pachyrhynchuspseudoproteus* Schultze, 1922**

**Type depository.**SMTD (syntypes).

**Type locality.** Philippines (Luzon Is.: Laguna Prov.).

**Distribution.** Philippines: Luzon Is. (Laguna) (Schultze 1922b; Schultze 1924).

**Remarks.** Two specimens examined by Rukmane (2019c) were recorded from Leyte Island (Baybey). However, the distribution of the other specimens was in Luzon and so requires further verification.

***Pachyrhynchuspsittacinus* Heller, 1912**

**Type depository.**SMTD (holotype).

**Type locality.** Philippines (Luzon Is.: Bataan Prov., Lamao).

**Distribution.** Philippines: Luzon Is. (Bataan, Lamao; Mountain) (Schultze 1924; Rukmane-Bārbale and Cabras 2021).

***Pachyrhynchuspsittaculus* Heller, 1921**

**Type depository.** Unknown.

**Type locality.** Philippines (Luzon Is.: Quezon Prov., Mt. Banahaw).

**Distribution.** Philippines: Luzon Is. (Laguna; Quezon) (Schultze 1924).

***Pachyrhynchuspulchellus* Behrens, 1887**

*Pachyrhynchusbakeri* Heller, 1921: 542. Synonymized by Schultze (1924: 338).

Pachyrhynchuspulchellusvar.modestioroides Schultze, 1922b: 576. Synonymized by Schultze (1924: 338).

**Type depository.**SMTD (*).

**Type locality.** Philippines.

**Distribution.** Philippines: Luzon Is. (Benguet, Baguio, Mt. Polis, Mt. Santo Tomas, Trinidad, Mt. Trail, Atoc; Bulacan, Guinnisan; Ifugao; Mountain Prov.; Nueva Vizcaya; Manila) (Heller 1921; Schultze 1922b, 1924; Rukmane 2019b, 2019c; Rukmane-Bārbale and Cabras 2021).

***Pachyrhynchusrebus* Rukmane, 2016**

**Type depository.**DUBC (holotype).

**Type locality.** Philippines (Luzon Is.: Quirino Prov., Nagtipunan).

**Distribution.** Only known from the type locality.

***Pachyrhynchusregiusregius* Schultze, 1922**

**Type depository.**SMTD (syntypes).

**Type locality.** Philippines (Leyte Is.: Cabalian).

**Distribution.** Philippines: Leyte Is. (mountains near Cabalian, Sogod); Mindanao Is. (Agusan del Norte, Sibagat; Bukidnon, Mt. Kiamo, Cabanglasan, San Fernando); Samar Is. (Marabot) (Schultze 1922b; Schultze 1923; Cabras et al. 2017; Rukmane-Bārbale and Cabras 2021).

***Pachyrhynchusregiusboronganus* Schultze, 1934**

**Type depository.**SMTD (syntypes).

**Type locality.** Philippines (Samar Is.: Eastern Samar, Borongan).

**Distribution.** Philippines: Samar Is. (​​Northern Samar; Samar; Eastern Samar, Borongan).

***Pachyrhynchusreicherti* Schultze, 1929**

**Type depository.**SMTD (holotype).

**Type locality.** Philippines (Mindanao Is.: Davao Prov., Mt. Apo).

**Distribution.** Philippines: Mindanao Is. (Bukidnon; Cotabato; Davao del Sur, Mt. Apo); Mindoro Is. (Schultze 1929; Cabras and Medina 2018; Rukmane 2019b; Rukmane-Bārbale and Cabras 2021).

***Pachyrhynchusreticulatus* Waterhouse, 1841**

**Type depository.**NHRS (syntype); SMTD (*).

**Type locality.** Philippines.

**Distribution.** Philippines: Catanduanes Is.; Luzon Is. (Abra; Aurora; Camarines Sur; Laguna, Lilio, Paete, Mt. Bonahao; Manila); Marinduque Is. (Boac) (Waterhouse 1841; Schultze 1923; Rukmane 2018c, 2019b, 2019c; Rukmane-Bārbale and Cabras 2021).

***Pachyrhynchusrizali* Schultze, 1934**

**Type depository.**SMTD (syntypes).

**Type locality.** Philippines (Luzon Is.: Aurora Prov., Casiguran; Nueva Vizcaya Prov., Mount Dibago).

**Distribution.** Philippines: Luzon Is. (Aurora, Casiguran; Cagayan, Gonzaga, Sta. Ana; Nueva Vizcaya, Mount Dibago; Quirino) (Rukmane-Bārbale and Cabras 2021; Villegas et al. 2024).

***Pachyrhynchusrochaorum* Yoshitake, 2020**

**Type depository.**NIAES (holotype, paratypes); KUM (paratypes).

**Type locality.** Philippines (Luzon Is.: Calabarzon region, Quezon Prov., Dolores, Brgy. Kinabuhayan, Mt. Banahaw).

**Distribution.** Only known from the type locality.

***Pachyrhynchusroseomaculatus* Waterhouse, 1841**

*Pachyrhynchusstriatus* Waterhouse, 1841: 25. Synonymized by Yoshitake (2019b: 193).

**Type depository.**NHMUK (holotype).

**Type locality.** Philippines.

**Distribution.** Philippines: Masbate Is. (Yoshitake 2019g).

**Remarks.** The specimen received by Schultze was obtained through an exchange, and he suggested that its location was Luzon Island (Schultze 1923). The only confirmed locality is Masbate Island, as examined by Yoshitake (2019g). Further research is needed to determine the distribution of this species.

***Pachyrhynchusrufopunctatus* Waterhouse, 1842**

**Type depository.**NHMUK (holotype).

**Type locality.** Philippines.

**Distribution.** Philippines: Luzon Is. (Manila); Mindoro Is. (Mt. Halcon); Polillo Is. (Waterhouse 1842; Schultze 1924; Rukmane 2018c; Rukmane-Bārbale and Cabras 2021).

***Pachyrhynchusrugicollisrugicollis* Waterhouse, 1841**

**Type depository.** Unknown.

**Type locality.** Philippines.

**Distribution.** Philippines: Luzon Is. (Aurora; Ifugao; Nueva Vizcaya, Sorsogon, Bulusan; Zambales Prov.; Battan Prov.; mountains of Mariveles; Manila) (Waterhouse 1841; Schultze 1923; Rukmane 2018c; Rukmane-Bārbale and Cabras 2021).

***Pachyrhynchusrugicollisaurinius* Heller, 1921**

**Type depository.** Unknown.

**Type locality.** Philippines (Luzon Is.: Zambales Prov., Iba).

**Distribution.** Only known from the type locality.

***Pachyrhynchusrugicolliscrucifer* Heller, 1912**

**Type depository.** Unknown.

**Type locality.** Philippines (Luzon Is.).

**Distribution.** Only known from the type locality.

***Pachyrhynchusrukmaneaerukmaneae* Barševskis, 2016**

*Pachyrhynchustakakuwai* Yoshitake, 2016b: 36. Synonymized by Yoshitake (2017c: 262).

**Type depository.**DUBC (holotype, paratypes).

**Type locality.** Philippines (Marinduque Is.: Boac).

**Distribution.** Philippines: Marinduque Is. (Buenavista, Mt. Malindig) (Barševskis 2016; Rukmane 2019b).

**Remarks.**Barševskis (2016) described this species based on a series of specimens from Marinduque Island, Philippines, including several other localities (e.g., Boac, Buenavista, and Mt. Malindig). However, Yoshitake and Yap (2017) considered that all materials of the species were collected from mountain areas, such as Sihi or Mt. Malindig, and suggested that the holotype locality, Boac, was mislabeled due to commercial processes. Yoshitake and Yap (2017) recommended that further studies were needed to clarify the distribution range of this species.

***Pachyrhynchusrukmaneaepaucisignatus* Yoshitake, 2017**

**Type depository.** KUM (holotype, paratypes); NIAES (paratypes).

**Type locality.** Philippines (Marinduque Is.: Boac).

**Distribution.** Philippines: Marinduque Is. (Boac) (Yoshitake and Yap 2017; Rukmane-Bārbale and Cabras 2021).

***Pachyrhynchussagittatus* Rukmane, 2019**

**Type depository.**DUBC (holotype).

**Type locality.** Philippines (Luzon Is.: Quirino Prov., Sierra Madre).

**Distribution.** Philippines: Luzon Is. (Quirino) (Rukmane 2019d, Rukmane-Bārbale and Cabras 2021).

***Pachyrhynchussakaii* Yoshitake, 2017**

**Type depository.**NIAES (holotype).

**Type locality.** Philippines (Samar Is.).

**Distribution.** Only known from the type locality (Yoshitake 2017c).

***Pachyrhynchussamarensis* Schultze, 1923**

**Type depository.** Unknown.

**Type locality.** Philippines (Samar Is.: Catarman).

**Distribution.** Philippines: Samar Is. (Northern Samar, Catarman, Lope De Vega; Samar) (Rukmane 2019a).

***Pachyrhynchussanchezi* Heller, 1912**

**Type depository.**SMTD (holotype).

**Type locality.** Philippines (Luzon Is.: Benguet Prov., Baguio).

**Distribution.** Philippines: Luzon Is. (Auror; Benguet, Baguio, Mt. Mirador, Mt. Santo Tomas, Mt. Trail; Ifugao; Isabela; Mountain Prov.; Nueva Vizcaya) (Schultze 1924; Heller 1912; Rukmane-Bārbale and Cabras 2021).

**Remarks.** Two specimens examined by Rukmane (2019b) were recorded from Mindanao Island (Surigao). However, other specimens were found in Luzon. It is likely that the two Mindanao specimens were mislabeled.

***Pachyrhynchussarcitissarcitis* Behrens, 1887**

**Type depository.** Unknown.

**Type locality.** Philippines.

**Distribution.** Philippines: Babuyan Islands. (Calayan Is.; Camiguin Is.; Fuga Is; Babuyan Is.); Batanes Islands (Batan Is.; Sabtang Is.; Itbayat Is.); Luzon Is. (Cagayan; Isabela; Quirino) (Schultze 1924; Rukmane 2019b; Rukmane-Bārbale and Cabras 2021; Tseng et al. 2021).

**Host plants.** Philippine Leea, *Leeaphilippinensis* (Leeaceae) and Manila Leea, *Leeaguineensis* (Leeaceae) (Tseng et al. 2021).

***Pachyrhynchussarcitiskotoensis* Kôno, 1930**

**Type depository.**HUMS (syntypes).

**Type locality.** Taiwan (Taitung County, Lanyu Is. (= Kotosho)).

**Distribution.** Taiwan: Lanyu Is.; Ludao Is.

**Host plants.** Philippine Leea, *Leeaphilippinensis* (Leeaceae) and Manila Leea, *Leeaguineensis* (Leeaceae) (this study).

**Remarks.** In a captive environment, the number of larval stages ranged from four to six, and the duration from eggs to adults varied between 133–169 days (Huang et al. 2018). A larva was found inside the stem of *L.guineensis* in Lanyu (this study). This species is relatively rarer on Ludao Is. than on Lanyu Is. (this study).

***Pachyrhynchusschoenherri* Waterhouse, 1841**

**Type depository.**NHMUK (holotype).

**Type locality.** Philippines.

**Distribution.** Philippine: Leyte Is. (Mahaplag) (Waterhouse 1841; Rukmane-Bārbale and Cabras 2021).

***Pachyrhynchusschuetzei* Schultze, 1917**

**Type depository.**SMTD (syntypes).

**Type locality.** Philippines (Luzon Is.: Benguet Prov., Haight’s Place).

**Distribution.** Philippines: Luzon Is. (Benguet, Loo, Mt. Pawai; Ifugao; Mountain Prov.; Nueva Vizcaya) (Schultze 1924; Rukmane 2019c; Rukmane-Bārbale and Cabras 2021).

***Pachyrhynchussemiignitus* Schultze, 1922**

**Type depository.**SMTD (holotype).

**Type locality.** Philippines (Mindanao Is.: Cotabato Prov., Pikit).

**Distribution.** Only known from the type locality (Schultze 1922b).

***Pachyrhynchussemperi* Heller, 1912**

**Type depository.**SMTD (syntypes).

**Type locality.** Philippines.

**Distribution.** Philippines: Babuyan Is. (Camiguin Norte; Calayan Is.; Babuyan Is.); Batanes Is. (Batan Is.) (Rukmane 2019b; Rukmane-Bārbale 2020b; Rukmane-Bārbale and Cabras 2021; Tseng et al. 2021).

**Host plants.** Bishop Wood Tree, *Bischofiajavanica* (Euphorbiaceae) (Tseng et al. 2021).

***Pachyrhynchusseptentrionalis* Yoshitake, 2017**

**Type depository.**NIAES (holotype, paratypes); CFS, KUM, MBLI (paratypes).

**Type locality.** Philippines (Luzon Is.: Cagayan Prov., Sierra Madre Mountains).

**Distribution.** Philippines: Luzon Is. (Cagayan Vally region, Aurora, Cagayan) (Yoshitake 2017d; Rukmane-Bārbale and Cabras 2021).

***Pachyrhynchussergejevae* Rukmane, 2018**

**Type depository.**DUBC (holotype, paratypes).

**Type locality.** Philippines (Mindanao Is.: Bukidnon Prov., Cabanglasan).

**Distribution.** Philippines: Mindanao Is. (Bukidnon, Mt. Kalatungan, Intavas) (Rukmane 2018f).

***Pachyrhynchusshavrini* Rukmane & Barševskis, 2016**

**Type depository.**DUBC (holotype, paratypes).

**Type locality.** Philippines (Samar Is.: Northern Samar Prov., Lope De Vega).

**Distribution.** Philippines: Samar Is. (Northern Samar, Lope De Vega; Samar, Hinabangan) (Rukmane-Bārbale and Cabras 2021).

**Remarks.** One specimen examined by Rukmane (2019b) was recorded from Luzon Island. However, the distribution of other specimens was in Samar. The distribution of *P.shavrini* requires further verification.

***Pachyrhynchussignaticollis* Schultze, 1922**

*Pachyrhynchustransversarius* Heller, 1923b: 8. Synonymized by Schultze (1923: 666).

**Type depository.**SMTD (syntypes).

**Type locality.** Philippines (Mindanao Is.: Agusan Prov., Butuan).

**Distribution.** Philippines: Mindanao Is. (Agusan Prov., Butuan; Surigao) (Schultze 1922a; Schultze 1923; Rukmane-Bārbale and Cabras 2021).

***Pachyrhynchussignatus* Schultze, 1919**

**Type depository.**SMTD (holotype).

**Type locality.** Philippines (Siargao Is.).

**Distribution.** Only known from the type locality.

***Pachyrhynchussimilis* Bollino, 2022**

**Type depository.**SMTD (holotype); MBLI, DUBC, CFS (paratypes).

**Type locality.** Philippines (Mindanao Is.: Lanao del Sur Prov., Near Wao).

**Distribution.** Philippines: Mindanao Is. (south eastern Lanao del Sur; north eastern North Cotabato; Bukidnon, west of Mount Kitanglad).

***Pachyrhynchussmaragdinus* Behrens, 1887**

Pachyrhynchussmaragdinusvar.carnosus Kraatz, 1888: 32. Synonymized by Schultze (1924: 313).

Pachyrhynchussmaragdinusvar.purpurascens Kraatz, 1888: 32. Synonymized by Schultze (1924: 313).

**Type depository.**SMTD (syntype).

**Type locality.** Philippines.

**Distribution.** Philippines: Bohol Is.; Mindanao Is. (Bukidnon, Misamis, Surigao); Samar Is. (Northern Samar, Lope De Vega; Samar, Hinabangan) (Rukmane 2019b; Rukmane-Bārbale and Cabras 2021).

***Pachyrhynchussonani* Kôno, 1930**

**Type depository.**HUMS (holotype).

**Type locality.** Taiwan (Taitung County, Lanyu Is. (= Kotosho)).

**Distribution.** Taiwan: Lanyu Is.

**Host plants.** Among all the individuals found on the host plants, 93.75% were found on Sea poison tree (*Barringtoniaasiatica*, Lecythidaceae), 3.13% on beef wood (*Casuarinaequisetifolia*, Casuarinaceae) and 3.13% on Indian almond (*Terminalia catappa*, Combretaceae) (Chen et al. 2017).

**Remarks.** One female was observed to lay eggs in the xylem of *B.asiatica*, and the larvae were also found in the decayed xylem of the same host plant (Hsu et al. 2017).

***Pachyrhynchusspeciosus* Waterhouse, 1841**

*Pachyrhynchusabsurdus* Schultze, 1919: 550. Synonymized by Schultze (1923: 657) and Rukmane-Bārbale (2020a: 35).

**Type depository.**NHMUK (syntype), NHRS (syntype), SMTD (syntypes).

**Type locality.** Philippines.

**Distribution.** Philippines: Bohol Is. (Bilar); Bucas Grande Is.; Dinagat Is.; Leyte Is.; Mindanao Is. (Bukidnon, Cabanglasan; Cotabato, Saob; Davao del Sur, Mt. Apo, Marilog District, Catigan, Toril, Davao City; Sarangani, Kiamba; Surigao Prov., Surigao, Tandag); Samar Is. (Northern Samar; Samar, Hinabangan); Siargao Is. (Waterhouse 1841; Schultze 1919, 1923; Háva and Rukmane 2018; Rukmane 2019a, b; Rukmane-Bārbale and Cabras 2021; Cabras et al. 2022a).

**Host plants.** This species was collected on the leaves of *Melastomamalabathricum* (Melastomataceae) and *Medinillacumingii* (Melastomataceae) (Cabras et al. 2022a).

***Pachyrhynchussphaericollaris* Schultze, 1923**

**Type depository.**SMTD (holotype).

**Type locality.** Philippines (Luzon Is.: Kalinga Prov., Pinukpuk).

**Distribution.** Philippines: Luzon Is. (Cagayan; Isabela, Pinablanca; Kalinga) (Rukmane 2019c; Rukmane-Bārbale and Cabras 2021).

***Pachyrhynchussphenomorphoides* Yoshitake, 2012**

**Type depository.**NIAES (holotype, paratypes).

**Type locality.** Philippines (Basilan Is.: near Zamboanga Peninsula of West Mindanao).

**Distribution.** Philippines: Basilan Is.; Mindanao Is. (Zamboanga del Norte Prov.) (Bollino 2022).

***Pachyrhynchusstellio* Heller, 1912**

**Type depository.**SMTD (syntypes).

**Type locality.** Philippines (Luzon Is.: Bataan Prov., Lamao).

**Distribution.** Philippines: Luzon Is. (Aurora, Bataan Prov., Lamao, Mt. Mariveles; Benguet, Isabela, Mountain, Nueva Vizcaya, Rizal) (Schultze 1923; Rukmane-Bārbale and Cabras 2021).

***Pachyrhynchussubamabilis* Yoshitake, 2012**

**Type depository.**NIAES (holotype).

**Type locality.** Philippines (Mindanao Is.: Mt. Apo).

**Distribution.** Philippines: Mindanao Is. (Bukidnon; Cotabato; Lanao del Sur) (Rukmane 2019b; Rukmane-Bārbale and Cabras 2021).

***Pachyrhynchussubanon* Bollino, 2022**

**Type depository.**SMTD (holotype); MBLI (paratypes).

**Type locality.** Philippines (Mindanao Is.: Misamis Occidental Prov., Brgy. Gandawan, Mt. Malindang Range, Lake Duminagat-Mt. Ginlajan).

**Distribution.** Only known from the type locality.

***Pachyrhynchussubgo* Bollino, 2025**

**Type depository.**SMTD (holotype); MBLI, DHA (paratypes).

**Type locality.** Philippines (Cebu Is.: Cebu Prov., Mt. Manunggal).

**Distribution.** Philippines: Cebu Is. (Cebu Prov., Balamban, Mt. Manunggal, Brgy. Sunog, Mt. Mauyog, hills north-east of Kaluangan) (Bollino and Ersoy 2025).

***Pachyrhynchussubpalidius* Rukmane-Bārbale, 2022**

**Type depository.**DUBC (holotype, paratypes).

**Type locality.** Philippines (Luzon Is.: Mountain Prov., Barlig, Bontoc).

**Distribution.** Only known from the type locality.

***Pachyrhynchussulphureomaculatus* Schultze, 1922**

**Type depository.**SMTD (holotype).

**Type locality.** Philippines (Mindanao Is.: Cotabato Prov., Cotabato).

**Distribution.** Philippines: Mindanao Is. (Agusan; Bukidnon, Mt. Kiamo; Cotabato, Kidapawan, Mt. Parker; Davao City, Marilog District, Mt. Apo; Sarrangani, Kiamba; Surigao del Sur, Esperanza, San Miguel, Tandag); Leyte Is. (Sogod); Samar Is. (Hinabangan) (Schultze 1922b; Cabras et al. 2017, 2022a; Rukmane-Bārbale and Cabras 2021).

**Host plant.***Lithocarpusboholensis* (Fagaceae) (Cabras et al. 2022a).

***Pachyrhynchussumptuosoides* Yoshitake, 2017**

**Type depository.** KUM (holotype, paratypes), NIAES (paratypes).

**Type locality.** Philippines (Luzon Is.: Apayao Prov., Conner).

**Distribution.** Philippines: Luzon Is. (Cordillera Administrative Region; Apayao, Conner; Cagayan, Santa Ana; Kalinga, Pinukpuk, Laguna) (Yoshitake 2017c; Rukmane-Bārbale 2020b; Rukmane-Bārbale and Cabras 2021).

***Pachyrhynchussumptuosus* Schultze, 1917**

**Type depository.**SMTD (syntypes).

**Type locality.** Philippines (Luzon Is.: Mountain Prov., Bontoc).

**Distribution.** Philippines: Luzon Is. (Apayao; Ilocos Sur, Cabagao; Kalinga, Lubuagan, Balbalan; Mountain Prov., Bontoc; Nueva Vizcaya) (Schultze 1924; Rukmane 2019c; Rukmane-Bārbale and Cabras 2021).

***Pachyrhynchustadauchii* Yoshitake, 2012**

**Type depository.**NIAES (holotype, paratypes).

**Type locality.** Philippines (Mindanao Is.: Surigao del Sur Prov., Bislig).

**Distribution.** Philippines: Mindanao Is. (Agusan; Agusan del Sur, San Francisco; Bukidnon, Cabanglasan; Davao del Sur, Mt. Apo, Mt. Matutum; Sarangani, Kiamba, Malungon, Maitum; Surigao del Sur, San Miguel, Tandag) (Rukmane 2019a; Rukmane-Bārbale and Cabras 2021).

***Pachyrhynchustayloritaylori* Schultze, 1922**

**Type depository.**SMTD (syntypes).

**Type locality.** Philippines (Luzon Is.: Kalinga Prov., Balbalan).

**Distribution.** Philippines: Luzon Is. (Kalinga; Nueva Vizcaya; Ifugao) (Schultze 1922a; Schultze 1924; Rukmane-Bārbale and Cabras 2021).

***Pachyrhynchustaylorimetalescens* Schultze, 1924**

**Type depository.**SMTD (syntypes).

**Type locality.** Philippines (Luzon Is.: Ifugao Prov., Polis Pass).

**Distribution.** Only known from the type locality.

***Pachyrhynchustetramaculatus* Rukmane, 2019**

**Type depository.**DUBC (holotype, paratypes).

**Type locality.** Philippines (Luzon Is.: Nueva Vizcaya Prov., Quezon).

**Distribution.** Only known from the type locality (Rukmane 2019d).

***Pachyrhynchustikoi* Rukmane, 2016**

**Type depository.**DUBC (holotype, paratypes).

**Type locality.** Philippines (Mindanao Is.: Bukidnon Prov., Cabanglasan).

**Distribution.** Philippines: Mindanao Is. (Bukidnon, Mt. Apo, Cabanglasan) (Rukmane-Bārbale and Cabras 2021).

***Pachyrhynchustilikensis* Bollino & Sandel, 2015**

**Type depository.**SMTD (holotype); MBLI, CFS (paratypes).

**Type locality.** Philippines (Lubang Is.: south-west of Tilik).

**Distribution.** Only known from the type locality.

***Pachyrhynchustobafolius* Kano, 1929**

**Type depository.** Unknown.

**Type locality.** Taiwan (Taitung County, Lanyu Is. (= Kotosho)).

**Distribution.** Taiwan: Lanyu Is., Laudao Is.

**Host plants.***Pipturusarborescens* (Urticaceae).

**Remarks.***Pachyrhynchustobafolius* is only distributed on Lanyu and Ludao islands. However, according to Rukmane (2019b), examination of specimens from NHRS revealed that two specimens were mislabeled with the collection site listed as Japan. Kano (1929) mentioned that *P.tobafolius* feeds on the leaves of *Barringtoniaasiatica* on the seashore. However, during our field observations, we found that this species mainly feeds on plants of the Urticaceae family (this study).

***Pachyrhynchustorresi* Rukmane, 2018**

**Type depository.**DUBC (holotype, paratypes).

**Type locality.** Philippines (Mindanao Is.: Zamboanga Prov., Labuan).

**Distribution.** Philippines: Mindanao Is. (Zamboanga, Labuan) (Rukmane 2018f, 2019b; Rukmane-Bārbale and Cabras 2021).

***Pachyrhynchustristis* Heller, 1912**

**Type depository.**NHMUK (syntypes), SDEI (syntypes), SMTD (syntypes).

**Type locality.** Philippines (Luzon Is.).

**Distribution.** Philippines: Luzon Is. (Benguet; Moutain Prov.) (Schultze 1924; Rukmane 2019b; Rukmane-Bārbale 2020b).

***Pachyrhynchusvalainisi* Rukmane & Barševskis, 2016**

**Type depository.**DUBC (holotype, paratype).

**Type locality.** Philippines (Mindoro Is.: Mindoro Oriental Prov., Puerto Galera).

**Distribution.** Philippines: Mindoro Is. (Mindoro Oriental Prov., Puerto Galera, Baco, Mt. Halcon) (Rukmane-Bārbale and Cabras 2021).

***Pachyrhynchusvenustusvenustus* Waterhouse, 1841**

Pachyrhynchusvenustusvar.aureomaculatus Kraatz, 1888: 27. Synonymized by Schultze (1924: 310).

*Pachyrhynchusvirgatus* Schultze, 1919: 549. Synonymized by Schultze (1924: 310).

**Type depository.**NHMUK (syntype), SMTD (syntypes).

**Type locality.** Philippines.

**Distribution.** Philippines: Mindanao Is. (Cotabato; Surigao Prov., Surigao; Sarangani); Samar Is. (Northern Samar, Lope De Vega; Samar, Hinabangan, Marabot); Leyte Is. (Sogod) (Waterhouse 1841; Schultze 1919; Rukmane-Bārbale and Cabras 2021).

**Remarks.** Six specimens examined by Rukmane (2019b) were recorded from Luzon Island (Manila). However, other specimens were found from Mindanao PAIC and the distribution of *P.venustus* requires further verification.

***Pachyrhynchusvenustusinsulanus* Schultze, 1919**

**Type depository.**SMTD (syntypes).

**Type locality.** Philippines (Siargao Is.; Bucas Grande Is.).

**Distribution.** Only known from the type locality.

***Pachyrhynchusviridans* Heller, 1912**

**Type depository.**SMTD (holotype).

**Type locality.** Philippines (Calayan).

**Distribution.** Philippines: Calayan Is.; Negros Is.; Sibuyan Is. (Heller 1912; Schultze 1924; Rukmane 2019b).

***Pachyrhynchusviridis* (Chevrolat, 1879)**

*Apocyrtusviridis* Chevrolat, 1879: 134.

*Sphenomorphaviridis*: Dalla Torre et al., 1931: 19.

*Pachyrhynchusviridis*: Yoshitake, 2017a: 243.

**Type depository.**NHRS (holotype).

**Type locality.** Indonesia (Dorey).

**Distribution.** Indonesia: Dorey Is.; West Papua (Manokwari Prov., Ransiki District, Neney village) (Yoshitake 2017a; Bollino 2023).

***Pachyrhynchusyoshitakei* Bollino & Rukmane, 2020**

**Type depository.**SMTD (holotype); MBLI (paratypes).

**Type locality.** Philippines (Mindanao Is.: Misamis Occidental Prov., Oroquieta City, Mt. Malindang Range, Mt. Capole-Sebucal).

**Distribution.** Philippines: Mindanao Is. (Misamis Occidental, Mt. Malindang Range).

***Pachyrhynchusyoshitakeorum* Yoshitake, Bollino & Sandel, 2019**

**Type depository.**NIAES (holotype, paratypes); KUM, CFS, MBLI (paratypes).

**Type locality.** Philippines (Bohol Is.: Bohol Prov., Duero, Brgy. Payao, Sitio Pangpang).

**Distribution.** Philippines: Bohol Is.; Dinagat Is.; Cebu Is. (Rukmane 2019a; Yoshitake et al. 2019b; Rukmane-Bārbale and Cabras 2021; Bollino and Ersoy 2025).

**Host plants.** This species is often found on cacao (*Theobromacacao*, Malvaceae) and coffee (*Coffeaarabica*, Rubiaceae) trees (Yoshitake et al. 2019b).

***Pachyrhynchusyukae* Yoshitake, 2019**

**Type depository.**NIAES (holotype, paratypes); MCKUM (paratypes).

**Type locality.** Philippines (Luzon Is.: Quezon Prov., Dolores, Barangay Kinabuhayan, Sitio Bangkong, Kahoy).

**Distribution.** Only known from the type locality.

***Pachyrhynchusyuukae* Yoshitake, 2019**

**Type depository.**NIAES (holotype, paratypes).

**Type locality.** Philippines (Luzon Is.: Nueva Ecija Prov., Carranglan, Lohong).

**Distribution.** Only known from the type locality (Yoshitake 2019d).

***Pachyrhynchuszamboanganus* Yoshitake, 2012**

**Type depository.**NIAES (holotype).

**Type locality.** Philippines (Mindanao Is.: Zamboanga del Norte Prov.).

**Distribution.** Only known from the type locality.

***Pachyrhynchuszebra* Schultze, 1917**

**Type depository.**SMTD (holotype).

**Type locality.** Philippines (Luzon Is.: Benguet Prov., Mt. Santo Tomas).

**Distribution.** Philippines: Luzon Is. (Aurora; Benguet, Baguio, Mt. Santo Tomas; Rizal) (Schultze 1923; Rukmane 2019b; Rukmane-Bārbale and Cabras 2021).

## ﻿Discussion

As the number of described *Pachyrhynchus* species has increased in recent years, it is vital to provide an updated checklist of species. Such compilations are integral to future faunistic studies as these lists can provide an overview of the species richness of a particular taxa with their natural history and distribution patterns. The present work provides an updated checklist of *Pachyrhynchus* species, including their host plants, geographic records, synonyms, type repositories, and species groups.

Compared to earlier works on *Pachyrhynchus* (e.g., Heller 1912; Schultze 1922a, 1923), recent publications incorporate host plant information (see Chen et al. 2017; Cabras et al. 2021, 2022a; Tseng et al. 2021). This information is crucial for understanding the feeding ecology of *Pachyrhynchus* species. In this account, 19 *Pachyrhynchus* species are provided with host plant records. Despite the limited number of records, they reflect the diversity of host plant taxa utilized by *Pachyrhynchus* species. A total of 26 families, 32 genera and 35 host plant species were recorded, including species from the genera *Ardisia* (Primulaceae), *Barringtonia* (Lecythidaceae), *Bischofia* (Euphorbiaceae), *Lithocarpus* (Fagaceae), *Melastoma* (Melastomataceae), *Philodendron* (Araceae), and *Saurauia* (Actinidiaceae) (Cabras and Yoshitake 2016; Chen et al. 2017; Hsu et al. 2017; Tseng et al. 2021; Cabras et al. 2022a). These relatively few records of host plant associations highlight significant knowledge gaps in *Pachyrhynchus* faunistic studies that need to be addressed.

We compared species group classifications of *Pachyrhynchus* weevils based on both morphological and molecular studies (Table 2). Morphology-based groupings were derived from three key sources: Heller (1912), Schultze (1923, 1924), and more recent taxonomic works published between 2017 and 2025. Molecular clades were based on Van Dam et al. (2023) and the phylogenetic framework from Chen et al. (2025). The comparison revealed that several morphological groups are consistent with molecular clades, suggesting concordance between traditional taxonomy and modern phylogenetic inference. For instance, *P.amabilis* was assigned to Group XIV by Schultze (1924) and to the *amabilis* species group in recent morphological taxonomy (Bollino et al. 2017, 2020), which aligns with the *amabilis* clade in Chen et al. (2025). Similarly, *P.anellifer* and *P.annulatus* consistently fall under Group VII (Heller 1912; Schultze 1924), and both are placed in the *erichsoni* clade based on molecular data (Van Dam et al. 2023; Chen et al. 2025). The *orbifer* clade, as defined in Chen et al. (2025), includes at least 19 species, such as *P.orbifer*, *P.cruciatus*, *P.jugifer*, *P.moniliferus*, *P.speciosus*, and *P.regius*. Most of these species were assigned to Group I or Group V based on the morphological classifications by Heller (1912) and Schultze (1923, 1924). This indicates a moderate level of consistency between traditional morphological groupings and recent molecular phylogenetic results. However, discrepancies were also noted. Some recently described species lack morphological group assignments in earlier literature but have been placed into molecular clades. Future studies focusing on a broader-scale comparison between morphological characters and molecular data are needed to clarify their taxonomic placement.

Distribution records are a vital element in *Pachyrhynchus* research, particularly regarding biogeography, insular speciation, and the evolution of mimicry systems. These records reveal spatial patterns such as co-occurrences and endemism. The Philippine archipelago is considered the center of diversity for the genus *Pachyrhynchus*, as the majority of described species are endemic to the country. Zoological demarcations of island groups in the Philippines are based on the Pleistocene Aggregate Island Complex (PAIC) model (Heaney 1986; Brown et al. 2013). Described *Pachyrhynchus* species in the Philippines primarily occur in the Greater Luzon Faunal Region and the Greater Mindanao Faunal Region. In the Greater Luzon Faunal Region, which encompasses mainland Luzon, Catanduanes, Polillo, Marinduque, and small surrounding islands, there are 82 known species. This region is the most species-rich, accounting for 45.8% of the described *Pachyrhynchus* species. Among these 82 species, 70 are endemic to this area. In the Greater Mindanao Faunal Region, which includes mainland Mindanao, Samar, Leyte, Biliran, Bohol, Dinagat, Siargao, Basilan, and small surrounding islands, there are 70 reported species, accounting for 39.1% of the described species (Fig. 2, Table 1), and 65 of them being endemic to this region.

**Table 1.** List of *Pachyrhynchus* species and their distribution regions. The letter ‘v’ indicates potential distribution areas mentioned in the literature. The abbreviations for distribution regions are as followed: (1) Greater Luzon PAIC (GL), (2) Greater Mindoro PAIC (GMDO), (3) Greater Negros-Panay PAIC (GNP), (4) Greater Mindanao PAIC (GMDA), (5) Lubang Island (LUB), (6) Sibuyan Island (SBY), (7) Islands north of mainland Luzon (INL), and (8) Indonesia archipelago (IND).

	Species	Distribution regions							
		(1) GL	(2) GMDO	(3) GNP	(4) GMDA	(5) LUB	(6) SBY	(7) INL	(8) IND
1	*P.amabilis* Schultze, 1922				v				
2	*P.anellifer* Heller, 1912	v							
3	*P.anichtchenkoi* Rukmane & Barševskis, 2016				v				
4	*P.annulatus* Chevrolat, 1881	v							
5	*P.antonkozlovi* Rukmane & Barševskis, 2016				v				
6	*P.apicatus* Schultze, 1922	v	v						
7	*P.apocyrtoides* Schultze, 1922				v				
8	*P.apoensis* Yoshitake, 2012				v				
9	*P.ardentius* Schultze, 1919				v				
10	*P.argus* Pascoe, 1873	v							
11	*P.atrocyaneus* Schultze, 1922				v				
12	*P.atronitens* Yoshitake, 2019	v							
13	*P.baluganus* Schultze, 1924	v							
14	*P.banglas* Bollino, Sandel & Rukmane, 2017				v				
15	*P.barsevskisi* Rukmane, 2016	v							
16	*P.basilanus* Heller, 1923				v				
17	*P.benguetanus* Schultze, 1924	v							
18	*P.bollinoi* Rukmane-Bārbale, 2020	v							
19	*P.bucasanus* Schultze, 1922				v				
	ssp.ornatus Schultze, 1934				v				
20	*P.cabrasae* Rukmane & Barševskis, 2016				v				
21	*P.caeruleovittatus* Yoshitake, 2012				v				
22	*P.caeruleus* Yoshitake, 2019	v							
23	*P.cagayanus* Heller, 1929	v							
24	*P.callainimaculatus* Yoshitake, 2017	v							
25	*P.cebrem* Patano & Rukmane-Bārbale, 2022				v				
26	*P.chamissoi* Schultze, 1922	v			v				
27	*P.chlorites* Chevrolat, 1881	v						v	
	P.chloritesssp.insularis Kano, 1929							v	
28	*P.chrysocyaneus* Rukmane-Bārbale, 2024	v							
29	*P.chrysomaculatus* Bollino, 2022				v				
30	*P.cinereomaculatus* Rukmane-Bārbale, 2020	v							
31	*P.cingulatus* Pascoe, 1873								v
32	*P.circulatus* Heller, 1912	v							
33	*P.circulimaculatus* Yoshitake, 2019				v				
34	*P.conformis* Yoshitake, 2017				v				
35	*P.confusus* Schultze, 1923	v							
36	*P.congestus* Pascoe, 1873	v							
	P.congestusssp.aedamlayroni Rukmane, 2019	v							
	P.congestusssp.coerulans Kraatz, 1888	v							
	P.congestusssp.immarginatus Kraatz, 1888	v							
	P.congestusssp.mirabilis Yoshitake, 2017	v							
	P.congestusssp.ocellatus Schultze, 1924	v							
	P.congestusssp.pavonius Heller, 1921	v							
37	*P.consobrinus* Schultze, 1922	v							
38	*P.corpulentus* Schultze, 1922				v				
	P.corpulentusssp.balatukan Patano & Macalaba, 2023				v				
39	*P.croesus* Oberthur, 1879								v
40	*P.cruciatus* Schultze, 1923	v							
41	*P.cumingii* Waterhouse, 1841				v				
	P.cumingiissp.boholensis Schultze, 1924				v				
42	*P.davaoensis* Schultze, 1934				v				
43	*P.decussatus* Waterhouse, 1841	v							
	P.decussatusssp.catanduanensis Rukmane-Bārbale, 2020	v							
44	*P.disargus* Rukmane, 2019	v							
45	*P.disgestus* Heller, 1929	v							
46	*P.dohrni* Behrens, 1887	v							
47	*P.domino* Rukmane, 2016		v						
48	*P.dubiosus* Schultze, 1922	v							
49	*P.elegans* Waterhouse, 1842				v				
50	*P.eques* Heller, 1912	v							
51	*P.equester* Heller, 1929	v							
52	*P.erichsoni* Waterhouse, 1841	v	v		v				
	P.erichsonissp.eschscholtzii Waterhouse, 1841	v							
53	*P.erosus* Schultze, 1920	v							
54	*P.esperanza* Bollino, Sandel & Rukmane, 2017				v				
55	*P.faisali* Bollino, 2023								v
56	*P.felipeae* Rukmane & Cabras, 2018			v					
57	*P.florulentus* Yoshitake, 2019	v							
58	*P.forsteni* Vollenhoven, 1864								v
59	*P.franciscoi* Rukmane & Cabras, 2018			v					
60	*P.galeraensis* Schultze, 1934		v						
61	*P.gemmatus* Waterhouse, 1841	v							
	P.gemmatusssp.purpureus Kraatz, 1888	v							
62	*P.gilvomaculatus* Yoshitake, 2017				v				
63	*P.gloriosus* Faust, 1895	v							
	P.gloriosusssp.abbreviatus Schultze, 1922	v							
64	*P.halconensis* Schultze, 1922		v						
65	*P.helenperrinae* Rukmane, 2018				v				
66	*P.helleri* Kuntzen, 1914	v							
67	*P.hirokii* Yoshitake, 2012				v				
68	*P.igorota* Schultze, 1917	v							
69	*P.ilgas* Rukmane, 2017				v				
70	*P.imitans* Rukmane & Bollino, 2020				v				
71	*P.inclytus* Pascoe, 1873	v							
72	*P.infernalis* Fairmaire, 1897							v	
73	*P.jitanasaius* Chen & Lin, 2017							v	
74	*P.jugifer* Waterhouse, 1841			v					
75	*P.kirklayroni* Rukmane, 2019	v							
76	*P.kraslavae* Rukmane & Barševskis, 2016				v				
77	*P.lacunosus* Heller, 1912	v							
78	*P.latifasciatus* Waterhouse, 1842				v				
79	*P.layroni* Rukmane & Cabras, 2018			v					
80	*P.libucanus* Schultze, 1923				v				
81	*P.loheri* Schultze, 1917	v							
82	*P.lorquini* Chevrolat, 1881	v							
83	*P.lubanganus* Bollino & Sandel, 2015					v			
84	*P.marinduquensis* Rukmane & Barševskis, 2016	v							
85	*P.maruyamai* Yoshitake, 2019	v							
86	*P.masatoshii* Yoshitake & Yap, 2017	v							
87	*P.masatoshiensis* Rukmane-Bārbale, 2024	v							
88	*P.miltoni* Cabras & Rukmane, 2016				v				
89	*P.mindoroensis* Rukmane & Háva, 2020		v						
90	*P.mohagani* Bollino & Sandel, 2015					v			
91	*P.möllendorffi* Heller, 1899			v					
	P.möllendorffissp.marinduquanus Rukmane & Cabras, 2018	v							
92	*P.moniliferus* Germar, 1824	v	v	v	v			v	
	P.moniliferusssp.abranus Heller, 1934	v	v						
	P.moniliferusssp.babuyanensis Rukmane, 2018							v	
	P.moniliferusssp.chevrolati Eydoux & Souleyet, 1839	v			v				
	P.moniliferusssp.herbidus Rukmane, 2018				v				
	P.moniliferusssp.jagori Heller, 1912				v				
	P.moniliferusssp.stellulifer Heller, 1912	v	v						
93	*P.morio* Heller, 1912	v							
94	*P.morotaiensis* Vollenhoven, 1864								v
95	*P.multipunctatus* Waterhouse, 1841	v			v				
	P.multipunctatusssp.endoi Yoshitake, 2018			v					
96	*P.naokii* Yoshitake, 2012				v				
97	*P.naokoae* Yoshitake, 2019						v		
98	*P.negrosensis* Schultze, 1924			v					
99	*P.neoabsurdus* Rukmane, 2017				v				
100	*P.niisatoi* Yoshitake, 2017	v							
101	*P.nitcisi* Rukmane & Barševskis, 2016				v				
102	*P.nobilis* Heller, 1912	v						v	
	P.nobilisssp.yamianus Kano, 1929							v	
103	*P.noeli* Yoshitake, 2019						v		
104	*P.notocruciatus* Yoshitake, 2017				v				
105	*P.obumanuvu* Cabras, Donato, Medina, & Van Dam, 2021				v				
106	*P.occidentalis* Rukmane, 2017				v				
107	*P.ochroplagiatus* Heller, 1912	v							
	P.ochroplagiatusssp.multiplagiatus Schultze, 1924	v							
108	*P.octoannulatus* Yoshitake, Bollino & Sandel, 2019				v				
109	*P.ohbayashii* Yoshitake, 2017								v
110	*P.orbifer* Waterhouse, 1841	v							
	P.orbiferssp.ardens Chevrolat, 1841	v							
	P.orbiferssp.azureus Schultze, 1922	v							
	P.orbiferssp.callainus Yoshitake, 2017	v							
	P.orbiferssp.circulifer Chevrolat, 1841	v							
	P.orbiferssp.gemmans Chevrolat, 1841	v							
	P.orbiferssp.inornatus Waterhouse, 1841	v						v	
	P.orbiferssp.murinus Heller, 1934	v							
	P.orbiferssp.striatomaculatus Yoshitake, 2017	v							
111	*P.orientalis* Rukmane, 2017				v				
112	*P.ottomerkli* Rukmane, 2019				v				
113	*P.panumanon* Cabras & Medina, 2022				v				
114	*P.perpulcher* Waterhouse, 1841	v						v	
115	*P.phaleratus* Waterhouse, 1841	v							
	P.phaleratusssp.badiovittatus Yoshitake, 2019	v							
	P.phaleratusssp.dannylayroni Rukmane, 2019	v							
116	*P.pinorum* Pascoe, 1873	v							
	P.pinorumssp.transversalis Heller, 1912	v							
117	*P.postpubescens* Schultze, 1922	v			v				
	P.postpubescensssp.confluens Janczyk, 1959				v				
118	*P.pseudamabilis* Yoshitake, 2012				v				
119	*P.pseudhalconensis* Rukmane, 2016		v						
120	*P.pseudoproteus* Schultze, 1922	v							
121	*P.psittacinus* Heller, 1912	v							
122	*P.psittaculus* Heller, 1921	v							
123	*P.pulchellus* Behrens, 1887	v							
124	*P.rebus* Rukmane, 2016	v							
125	*P.regius* Schultze, 1922				v				
	P.regiusssp.boronganus Schultze, 1934				v				
126	*P.reicherti* Schultze, 1929				v				
127	*P.reticulatus* Waterhouse, 1841	v							
128	*P.rizali* Schultze, 1934	v							
129	*P.rochaorum* Yoshitake, 2020	v							
130	*P.roseomaculatus* Waterhouse, 1841			v					
131	*P.rufopunctatus* Waterhouse, 1842	v	v						
132	*P.rugicollis* Waterhouse, 1841	v							
	P.rugicollisssp.aurinius Heller, 1921	v							
	P.rugicollisssp.crucifer Heller, 1912	v							
133	*P.rukmaneae* Barševskis, 2016	v							
	P.rukmaneaessp.paucisignatus Yoshitake, 2017	v							
134	*P.sagittatus* Rukmane, 2019	v							
135	*P.sakaii* Yoshitake, 2017				v				
136	*P.samarensis* Schultze, 1923				v				
137	*P.sanchezi* Heller, 1912	v							
138	*P.sarcitis* Behrens, 1887	v						v	
	P.sarcitisssp.kotoensis Kôno, 1930							v	
139	*P.schoenherri* Waterhouse, 1841				v				
140	*P.schuetzei* Schultze, 1917	v							
141	*P.semiignitus* Schultze, 1922				v				
142	*P.semperi* Heller, 1912							v	
143	*P.septentrionalis* Yoshitake, 2017	v							
144	*P.sergejevae* Rukmane, 2018				v				
145	*P.shavrini* Rukmane & Barševskis, 2016				v				
146	*P.signaticollis* Schultze, 1922				v				
147	*P.signatus* Schultze, 1919				v				
148	*P.similis* Bollino, 2022				v				
149	*P.smaragdinus* Behrens, 1887				v				
150	*P.sonani* Kôno, 1930							v	
151	*P.speciosus* Waterhouse, 1841				v				
152	*P.sphaericollaris* Schultze, 1923	v							
153	*P.sphenomorphoides* Yoshitake, 2012				v				
154	*P.stellio* Heller, 1912	v							
155	*P.subamabilis* Yoshitake, 2012				v				
156	*P.subanon* Bollino, 2022				v				
157	*P.subpalidius* Rukmane-Bārbale, 2022	v							
158	*P.sugbo* Bollino, 2025			v					
159	*P.sulphureomaculatus* Schultze, 1922				v				
160	*P.sumptuosoides* Yoshitake, 2017	v							
161	*P.sumptuosus* Schultze, 1917	v							
162	*P.tadauchii* Yoshitake, 2012				v				
163	*P.taylori* Schultze, 1922	v							
	P.taylorissp.metalescens Schultze, 1924	v							
164	*P.tetramaculatus* Rukmane, 2019	v							
165	*P.tikoi* Rukmane, 2016				v				
166	*P.tilikensis* Bollino & Sandel, 2015					v			
167	*P.tobafolius* Kano, 1929							v	
168	*P.torresi* Rukmane, 2018				v				
169	*P.tristis* Heller, 1912	v							
170	*P.valainisi* Rukmane & Barševskis, 2016		v						
171	*P.venustus* Waterhouse, 1841				v				
	P.venustusssp.insulanus Schultze, 1919				v				
172	*P.viridans* Heller, 1912			v			v	v	
173	*P.viridis* (Chevrolat, 1879)								v
174	*P.yoshitakei* Bollino & Rukmane, 2020				v				
175	*P.yoshitakeorum* Yoshitake, Bollino & Sandel, 2019			v	v				
176	*P.yukae* Yoshitake, 2019	v							
177	*P.yuukae* Yoshitake, 2019	v							
178	*P.zamboanganus* Yoshitake, 2012				v				
179	*P.zebra* Schultze, 1917	v							
Species		82	10	11	70	3	3	11	7
Subspecies		30	2	1	9	0	0	5	0
Total		112	12	12	79	3	3	16	7

**Table 2.** List of *Pachyrhynchus* species groups, including both morphology-based groups and molecular clades. *Pachyrhynchusabsurdus* was synonymized with *P.speciosus* by Rukmane-Bārbale (2020); however, the term “*absurdus* species group” is retained here in accordance with the original classification proposed in Rukmane 2017.

	Species	Morphology-based group			Molecular clade	
		Heller 1912	Schultze 1923, 1924	Various studies (2017–2022)	Van Dam et al. 2023	Chen et al. 2025
1	*P.amabilis* Schultze, 1922		Group XIV	*amabilis* species group		*amabilis* clade
2	*P.anellifer* Heller, 1912	Group VII	Group VII			*erichsoni* clade
3	*P.anichtchenkoi* Rukmane & Barševskis, 2016					*decussatus* clade
4	*P.annulatus* Chevrolat, 1881	Group VII	Group VII		*erichsoni* clade	*erichsoni* clade
5	*P.antonkozlovi* Rukmane & Barševskis, 2016					*erichsoni* clade
6	*P.apicatus* Schultze, 1922		Group V		*apicatus* clade	*gemmatus* clade
7	*P.apocyrtoides* Schultze, 1922		Group IV			
8	*P.apoensis* Yoshitake, 2012			*schoenherri* species group		*gemmatus* clade
9	*P.ardentius* Schultze, 1919		Group XII	*schoenherri* species group		
10	*P.argus* Pascoe, 1873	Group VII	Group XV		*argus* clade	*congestus* clade
11	*P.atrocyaneus* Schultze, 1922		Group XV	*atrocyaneus* species group		
12	*P.atronitens* Yoshitake, 2019					*pinorum* clade
13	*P.baluganus* Schultze, 1924		Group VII		*erichsoni* clade	
14	*P.banglas* Bollino, Sandel & Rukmane, 2017			*amabilis* species group		
15	*P.barsevskisi* Rukmane, 2016			*pinorum* species group	*lacunosus* clade	*pinorum* clade
16	*P.basilanus* Heller, 1923		Group XV	*atrocyaneus* species group		
17	*P.benguetanus* Schultze, 1924		Group VIII			
18	*P.bollinoi* Rukmane-Bārbale, 2020					*pinorum* clade
19	*P.bucasanus* Schultze, 1922		Group IV			
20	*P.cabrasae* Rukmane & Barševskis, 2016			*speciosus* species group		
21	*P.caeruleovittatus* Yoshitake, 2012				*amabilis* clade	*amabilis* clade
22	*P.caeruleus* Yoshitake, 2019					*congestus* clade
23	*P.cagayanus* Heller, 1929					*decussatus* clade
24	*P.callainimaculatus* Yoshitake, 2017			*pinorum* species group		
25	*P.cebrem* Patano & Rukmane-Bārbale, 2022					
26	*P.chamissoi* Schultze, 1922		Group XIV	*amabilis* species group		
27	*P.chlorites* Chevrolat, 1881	Group IV	Group VIII			*congestus* clade
28	*P.chrysocyaneus* Rukmane-Bārbale, 2024					
29	*P.chrysomaculatus* Bollino, 2022			*atrocyaneus* species group		
30	*P.cinereomaculatus* Rukmane-Bārbale, 2020					*pinorum* clade
31	*P.cingulatus* Pascoe, 1873					
32	*P.circulatus* Heller, 1912	Group V	Group I			*orbifer* clade
33	*P.circulimaculatus* Yoshitake, 2019			*speciosus* species group		
34	*P.conformis* Yoshitake, 2017					
35	*P.confusus* Schultze, 1923		Group V			
36	*P.congestus* Pascoe, 1873	Group IV	Group VIII		*congestus* clade	*congestus* clade
37	*P.consobrinus* Schultze, 1922		Group XIII	*pinorum* species group	*lacunosus* clade	*pinorum* clade
38	*P.corpulentus* Schultze, 1922		Group XII	*schoenherri* species group	*phaleratus* clade	*gemmatus* clade
39	*P.croesus* Oberthur, 1879	Group III	Group XV			*gemmatus* clade
40	*P.cruciatus* Schultze, 1923		Group I		*orbifer* clade	*orbifer* clade
41	*P.cumingii* Waterhouse, 1841	Group V	Group XI			*decussatus* clade
42	*P.davaoensis* Schultze, 1934			*speciosus* species group		*orbifer* clade
43	*P.decussatus* Waterhouse, 1841	Group V	Group I			*decussatus* clade
44	*P.disargus* Rukmane, 2019				*argus* clade	
45	*P.disgestus* Heller, 1929					
46	*P.dohrni* Behrens, 1887	Group II	Group VIII		*congestus* clade	*congestus* clade
47	*P.domino* Rukmane, 2016					
48	*P.dubiosus* Schultze, 1922		Group XIII	*pinorum* species group	*lacunosus* clade	
49	*P.elegans* Waterhouse, 1842	Group VI	Group VI	*schoenherri* species group	*phaleratus* clade	*gemmatus* clade
50	*P.eques* Heller, 1912	Group I	Group IX			*congestus* clade
51	*P.equester* Heller, 1929					
52	*P.erichsoni* Waterhouse, 1841	Group III	Group IV		*erichsoni* clade	*erichsoni* clade
53	*P.erosus* Schultze, 1920		Group VII		*erichsoni* clade	*erichsoni* clade
54	*P.esperanza* Bollino, Sandel & Rukmane, 2017			*schoenherri* species group		
55	*P.faisali* Bollino, 2023					
56	*P.felipeae* Rukmane & Cabras, 2018					*erichsoni* clade
57	*P.florulentus* Yoshitake, 2019					*pinorum* clade
58	*P.forsteni* Vollenhoven, 1864	Group I	Group III			
59	*P.franciscoi* Rukmane & Cabras, 2018					*erichsoni* clade
60	*P.galeraensis* Schultze, 1934					*erichsoni* clade
61	*P.gemmatus* Waterhouse, 1841	Group IV	Group VIII		*congestus* clade	*gemmatus* clade
62	*P.gilvomaculatus* Yoshitake, 2017					*amabilis* clade
63	*P.gloriosus* Faust, 1895	Group II	Group XI		*gloriosus* clade	*decussatus* clade
64	*P.halconensis* Schultze, 1922		Group I			*gemmatus* clade
65	*P.helenperrinae* Rukmane, 2018					
66	*P.helleri* Kuntzen, 1914		Group V			*gemmatus* clade
67	*P.hirokii* Yoshitake, 2012			*atrocyaneus* species group		*amabilis* clade
68	*P.igorota* Schultze, 1917		Group X			*decussatus* clade
69	*P.ilgas* Rukmane, 2017			*absurdus* species group		
70	*P.imitans* Rukmane & Bollino, 2020			*amabilis* species group		*amabilis* clade
71	*P.inclytus* Pascoe, 1873	Group II	Group X		*inclytus* clade	*decussatus* clade
72	*P.infernalis* Fairmaire, 1897	Group I	Group I			*orbifer* clade
73	*P.jitanasaius* Chen & Lin, 2017					*orbifer* clade
74	*P.jugifer* Waterhouse, 1841	Group V	Group I			*orbifer* clade
75	*P.kirklayroni* Rukmane, 2019					*decussatus* clade
76	*P.kraslavae* Rukmane & Barševskis, 2016			*speciosus* species group	*miltoni* clade	*orbifer* clade
77	*P.lacunosus* Heller, 1912	Group III	Group XIII	*pinorum* species group	*lacunosus* clade	*pinorum* clade
78	*P.latifasciatus* Waterhouse, 1842	Group V	Group II		*erichsoni* clade	*erichsoni* clade
79	*P.layroni* Rukmane & Cabras, 2018				*orbifer* clade	
80	*P.libucanus* Schultze, 1923		Group I			
81	*P.loheri* Schultze, 1917		Group X	*pinorum* species group		*pinorum* clade
82	*P.lorquini* Chevrolat, 1881	Group IV	Group VIII			*congestus* clade
83	*P.lubanganus* Bollino & Sandel, 2015					
84	*P.marinduquensis* Rukmane & Barševskis, 2016					*orbifer* clade
85	*P.maruyamai* Yoshitake, 2019					
86	*P.masatoshii* Yoshitake & Yap, 2017					
87	*P.masatoshiensis* Rukmane-Bārbale, 2024					
88	*P.miltoni* Cabras & Rukmane, 2016			*speciosus* species group	*miltoni* clade	
89	*P.mindoroensis* Rukmane & Háva, 2020				*congestus* clade	*congestus* clade
90	*P.mohagani* Bollino & Sandel, 2015					
91	*P.möllendorffi* Heller, 1899	Group II	Group X		*gloriosus* clade	*decussatus* clade
92	*P.moniliferus* Germar, 1824	Group V	Group I		*orbifer* clade	*orbifer* clade
93	*P.morio* Heller, 1912	Group IV	Group VIII			
94	*P.morotaiensis* Vollenhoven, 1864	Group I	Group III			*morotaiensis* clade
95	*P.multipunctatus* Waterhouse, 1841	Group VI	Group VI			
96	*P.naokii* Yoshitake, 2012			*atrocyaneus* species group		
97	*P.naokoae* Yoshitake, 2019					
98	*P.negrosensis* Schultze, 1924					
99	*P.neoabsurdus* Rukmane, 2017			*absurdus* species group		
100	*P.niisatoi* Yoshitake, 2017			*pinorum* species group	*lacunosus* clade	*pinorum* clade
101	*P.nitcisi* Rukmane & Barševskis, 2016			*schoenherri* species group		
102	*P.nobilis* Heller, 1912	Group II	Group XI			*erichsoni* clade
103	*P.noeli* Yoshitake, 2019					
104	*P.notocruciatus* Yoshitake, 2017			*speciosus* species group		*orbifer* clade
105	*P.obumanuvu* Cabras, Donato, Medina, & Van Dam, 2021					
106	*P.occidentalis* Rukmane, 2017			*absurdus* species group	*miltoni* clade	*orbifer* clade
107	*P.ochroplagiatus* Heller, 1912	Group I	Group XV		*argus* clade	*congestus* clade
108	*P.octoannulatus* Yoshitake, Bollino & Sandel, 2019			*speciosus* species group		*orbifer* clade
109	*P.ohbayashii* Yoshitake, 2017					
110	*P.orbifer* Waterhouse, 1841	Group V	Group I		*orbifer* clade	*orbifer* clade
111	*P.orientalis* Rukmane, 2017			*absurdus* species group		
112	*P.ottomerkli* Rukmane, 2019					
113	*P.panumanon* Cabras & Medina, 2022					
114	*P.perpulcher* Waterhouse, 1841	Group III	Group XV		*gloriosus* clade	*decussatus* clade
115	*P.phaleratus* Waterhouse, 1841	Group V	Group I		*phaleratus* clade	*gemmatus* clade
116	*P.pinorum* Pascoe, 1873	Group III	Group XIII	*pinorum* species group	*lacunosus* clade	*pinorum* clade
117	*P.postpubescens* Schultze, 1922		Group II	*speciosus* species group		
118	*P.pseudamabilis* Yoshitake, 2012			*amabilis* species group	*amabilis* clade	*amabilis* clade
119	*P.pseudhalconensis* Rukmane, 2016				*gloriosus* clade	*decussatus* clade
120	*P.pseudoproteus* Schultze, 1922		Group VI			
121	*P.psittacinus* Heller, 1912	Group III	Group X			*pinorum* clade
122	*P.psittaculus* Heller, 1921		Group X			*pinorum* clade
123	*P.pulchellus* Behrens, 1887	Group II	Group X		*gloriosus* clade	*decussatus* clade
124	*P.rebus* Rukmane, 2016					
125	*P.regius* Schultze, 1922		Group II	*speciosus* species group		*orbifer* clade
126	*P.reicherti* Schultze, 1929					*orbifer* clade
127	*P.reticulatus* Waterhouse, 1841	Group V	Group I		*miltoni* clade	*orbifer* clade
128	*P.rizali* Schultze, 1934					*erichsoni* clade
129	*P.rochaorum* Yoshitake, 2020					*decussatus* clade
130	*P.roseomaculatus* Waterhouse, 1841	Group IV	Group I			
131	*P.rufopunctatus* Waterhouse, 1842		Group V			
132	*P.rugicollis* Waterhouse, 1841	Group V	Group I		*orbifer* clade	*orbifer* clade
133	*P.rukmaneae* Barševskis, 2016				*congestus* clade	*congestus* clade
134	*P.sagittatus* Rukmane, 2019					
135	*P.sakaii* Yoshitake, 2017					
136	*P.samarensis* Schultze, 1923		Group II	*speciosus* species group		
137	*P.sanchezi* Heller, 1912	Group IV	Group VIII		*phaleratus* clade	*gemmatus* clade
138	*P.sarcitis* Behrens, 1887	Group IV	Group VIII			*gemmatus* clade
139	*P.schoenherri* Waterhouse, 1841	Group III	Group IV	*schoenherri* species group		*gemmatus* clade
140	*P.schuetzei* Schultze, 1917		Group VII			
141	*P.semiignitus* Schultze, 1922		Group IV			
142	*P.semperi* Heller, 1912	Group II	Group X	*pinorum* species group		*pinorum* clade
143	*P.septentrionalis* Yoshitake, 2017			*pinorum* species group	*lacunosus* clade	*pinorum* clade
144	*P.sergejevae* Rukmane, 2018			*atrocyaneus* species group		
145	*P.shavrini* Rukmane & Barševskis, 2016				*inclytus* clade	*decussatus* clade
146	*P.signaticollis* Schultze, 1922		Group IV			
147	*P.signatus* Schultze, 1919		Group IV			
148	*P.similis* Bollino, 2022			*atrocyaneus* species group		*amabilis* clade
149	*P.smaragdinus* Behrens, 1887	Group III	Group V		*apicatus* clade	
150	*P.sonani* Kôno, 1930					*orbifer* clade
151	*P.speciosus* Waterhouse, 1841	Group V	Group II	*speciosus* species group	*miltoni* clade	*orbifer* clade
152	*P.sphaericollaris* Schultze, 1923		Group I			
153	*P.sphenomorphoides* Yoshitake, 2012			*atrocyaneus* species group		*amabilis* clade
154	*P.stellio* Heller, 1912	Group V	Group I			
155	*P.subamabilis* Yoshitake, 2012			*amabilis* species group		*amabilis* clade
156	*P.subanon* Bollino, 2022			*atrocyaneus* species group		
157	*P.subpalidius* Rukmane-Bārbale, 2022					
158	*P.sugbo* Bollino, 2025			*moniliferus* species group		
159	*P.sulphureomaculatus* Schultze, 1922		Group V		*apicatus* clade	
160	*P.sumptuosoides* Yoshitake, 2017			*pinorum* species group		
161	*P.sumptuosus* Schultze, 1917		Group IX		*congestus* clade	*congestus* clade
162	*P.tadauchii* Yoshitake, 2012			*speciosus* species group		
163	*P.taylori* Schultze, 1922		Group VIII			*gemmatus* clade
164	*P.tetramaculatus* Rukmane, 2019					*pinorum* clade
165	*P.tikoi* Rukmane, 2016			*amabilis* species group		
166	*P.tilikensis* Bollino & Sandel, 2015					*gemmatus* clade
167	*P.tobafolius* Kano, 1929					*decussatus* clade
168	*P.torresi* Rukmane, 2018			*atrocyaneus* species group		
169	*P.tristis* Heller, 1912	Group III	Group XIII	*pinorum* species group		
170	*P.valainisi* Rukmane & Barševskis, 2016					
171	*P.venustus* Waterhouse, 1841	Group III	Group V			*gemmatus* clade
172	*P.viridans* Heller, 1912	Group IV	Group VIII			*congestus* clade
173	*P.viridis* (Chevrolat, 1879)					
174	*P.yoshitakei* Bollino & Rukmane, 2020			*amabilis* species group		
175	*P.yoshitakeorum* Yoshitake, Bollino & Sandel, 2019			*speciosus* species group		
176	*P.yukae* Yoshitake, 2019					
177	*P.yuukae* Yoshitake, 2019					*congestus* clade
178	*P.zamboanganus* Yoshitake, 2012			*amabilis* species group		*amabilis* clade
179	*P.zebra* Schultze, 1917		Group I			

The islands north of mainland Luzon, although consisting of only a small group of islands, harbor 11 species (6.2%), of which 4 are endemic. The island of Mindoro has ten species, representing 5.6% of the described species, with six being endemic. The Greater Negros-Panay Faunal Region has 11 species (8 of which are endemic), representing 6.1% of the described species (Fig. 2, Table 1). Additionally, oceanic islands such as Lubang and Sibuyan contain three species each, *P.lubanganus*, *P.mohagani*, and *P.tilikinesis* on Lubang; *P.noeli*, *P.naokoae*, and *P.viridans* on Sibuyan. Among them, only *P.viridans* is also distributed in the Greater Negros-Panay PAIC and the islands north of mainland Luzon. *Pachyrhynchus* species found in the Indonesia archipelago do not occur in the Philippines. Furthermore, 17 *Pachyrhynchus* species and subspecies are known to occur in more than one faunal region (Table 1).

Despite these records, many areas in the Philippines remain underexplored for *Pachyrhynchus*, such as the Bicol Peninsula in southern Luzon and the Greater Sulu Faunal Region, which encompasses the archipelagos of Sulu and Tawi-Tawi. Among the 179 *Pachyrhynchus* species, 165 species are endemic to specific regions or islands, indicating the high level of endemism (92.2%) in these weevils. The high endemism also underscores the importance of biodiversity conservation in these areas. In Taiwan, for example, three endemic species and three endemic subspecies are distributed on two small islands. Due to their endemism and high collection pressure, all the *Pachyrhynchus* species were listed as rare and valuable species protected under the Wildlife Conservation Act in 2009. In 2019, *Pachyrhynchus* species in the Philippines were listed as vulnerable in the national list of threatened Philippine fauna and their categories. Deforestation for agricultural purposes poses a threat to *Pachyrhynchus* weevils in the Philippines, potentially causing habitat loss, especially for locally endemic species (Cabras and Rukmane 2016). Continuously updating records of *Pachyrhynchus* will not only enhance our understanding of its patterns of diversity but also aid in identifying priority conservation sites for this genus, especially for threatened species.

## ﻿Additional information

### Author ORCIDs

Shan-Min Chen https://orcid.org/0009-0009-7346-0909

Ace Kevin S. Amarga https://orcid.org/0000-0002-9776-6576

Jing-Fu Tsai https://orcid.org/0000-0003-3076-3976

Bin-Hong Ho https://orcid.org/0000-0001-6008-9076

Hui-Yun Tseng https://orcid.org/0000-0002-1166-4756

### Data availability

All of the data that support the findings of this study are available in the main text.
